# Wavelet transforms on Gelfand-Shilov spaces and concrete examples

**DOI:** 10.1186/s13660-017-1393-0

**Published:** 2017-05-18

**Authors:** Naohiro Fukuda, Tamotu Kinoshita, Kazuhisa Yoshino

**Affiliations:** 1 Matsue College, National Institute of Technology, Matsue, Shimane 690-8518 Japan; 2 Institute of Mathematics, Tsukuba University, Tsukuba, Ibaraki 305-8571 Japan

**Keywords:** 42C10, 46F05, 65T60, wavelet transform, Gelfand-Shilov space, continuity properties

## Abstract

In this paper, we study the continuity properties of wavelet transforms in the Gelfand-Shilov spaces with the use of a vanishing moment condition. Moreover, we also compute the Fourier transforms and the wavelet transforms of concrete functions in the Gelfand-Shilov spaces.

## Introduction

In recent years, the wavelet transform has been shown to be a successful tool in signal processing applications such as data compression and fast computations. The wavelet transform of $f \in L^{2}(\mathbf{R})$ with respect to the analyzed wavelet $\psi\in L^{2}(\mathbf{R})$ satisfying the admissible condition $C_{\psi}:=\int_{\mathbf{R}} \vert \hat{\psi}(\xi) \vert ^{2} / \vert \xi \vert \,d\xi <\infty$ is defined by

$$W_{\psi}f (a,b) =\frac{1}{\sqrt{C_{\psi}}} \int_{\mathbf{R}}f(x) \overline{\psi_{a,b}(x)}\,dx, $$

where

$$\psi_{a,b}(x)=\frac{1}{\sqrt{ a }}\psi \biggl( \frac{x-b}{a} \biggr)\quad (a>0, b \in\mathbf{R}) $$

(see [1, 2] for example). The inverse wavelet transform of $F \in L^{2}(\mathbf{R}_{+}\times\mathbf{R})$ with respect to the analyzed wavelet $\psi\in L^{2}(\mathbf{R})$ is defined by

$$M_{\psi}F(x)=\frac{1}{\sqrt{C_{\psi}}} \int_{\mathbf{R}_{+}} \int_{\mathbf{R}} F(a,b) \psi_{a,b}(x) \frac{db\,da}{a^{2}}\quad (x \in\mathbf{R}). $$

For the time-frequency analysis, we are concerned with better localization in both time and frequency spaces from a point of view of the uncertainty principle. For the well-balanced localization, it would be suitable to consider the Schwartz space ${\mathcal {S}}$ regarded as the space of functions which have arbitrary polynomial decay and whose Fourier transforms also have arbitrary polynomial decay (see [3]). For instance, the typical Mexican hat wavelet belongs to spaces of more rapidly decreasing and more regular functions in ${\mathcal {S}}$. In this article we focus on Gelfand-Shilov spaces of functions which have sub-exponential decay and whose Fourier transforms also have sub-exponential decay. For positive constants *μ*, *ν* and *h* such that $\mu+\nu\geq 1$, we define the Banach Gelfand-Shilov space

$${\mathcal {S}}^{\mu}_{\nu, h}(\mathbf{R}) = \bigl\{ f \in{ \mathcal {S}} ; \bigl\Vert x^{\alpha}\partial_{x}^{\beta}f(x) \bigr\Vert _{L^{\infty}(\mathbf{R})} \leq C h^{\alpha+\beta }\alpha!^{\nu}\beta!^{\mu}\text{ for all } \alpha, \beta\in\mathbf{N} \bigr\} $$

with the norm

$$\Vert f \Vert _{{\mathcal {S}}^{\mu}_{\nu, h}(\mathbf{R})}= \sup_{\alpha ,\beta \in\mathbf{N}} \frac{ \Vert x^{\alpha}\partial_{x}^{\beta}f(x) \Vert _{L^{\infty}(\mathbf {R})} }{h^{\alpha+\beta }\alpha!^{\nu}\beta!^{\mu}}, $$

and the (non-Banach) Gelfand-Shilov space ${\mathcal {S}}^{\mu}_{\nu }(\mathbf{R}) $

$${\mathcal {S}}^{\mu}_{\nu}(\mathbf{R}) = \operatorname {ind}\lim _{h>0}{\mathcal {S}}^{\mu}_{\nu, h}(\mathbf{R}) $$

with the inductive limit topology. The Gelfand-Shilov spaces were originally introduced in [4] and [5]. As well explained in [6] and [7], the Gelfand-Shilov spaces are better adapted to the study of the problems of partial differential equations for which the solutions sub-exponentially decay at infinity.

### Remark 1.1

Restricting functions with Fourier transforms supported in the right half-plane, we may also define the Banach progressive Gelfand-Shilov space

$${\mathcal {S}}^{\mu,+}_{\nu,h}(\mathbf{R}) = \bigl\{ f \in{\mathcal {S}}^{\mu}_{\nu,h}(\mathbf{R}) ; \operatorname {supp}\hat{f} \subset[0, \infty) \bigr\} , $$

and the (non-Banach) progressive Gelfand-Shilov space

$${\mathcal {S}}^{\mu,+}_{\nu}(\mathbf{R}) = \bigl\{ f \in{\mathcal {S}}^{\mu}_{\nu}(\mathbf{R}) ; \operatorname {supp}\hat{f} \subset[0, \infty) \bigr\} . $$

Such spaces can be considered in dealing with analytic signals as the Hardy space $H^{2}(\mathbf{R})$ (see [8]). If the analyzed wavelet *ψ* belongs to the progressive Gelfand-Shilov space, *ψ̂* smoothly tends to zero and also has vanishing moments. For example, the Bessel wavelet $\psi(x)$ defined by $\hat{\psi}(\xi) = e^{-\xi- \xi^{-1}}$ for $\xi> 0$ and $\hat{\psi}(\xi) =0$ for $\xi\leq 0$ belongs to ${\mathcal {S}}^{1,+}_{2}(\mathbf{R})$. Actually, we know that $\psi(x)=\frac{1}{\pi\sqrt{1-ix}}K_{1}(2\sqrt{1-ix})$, where $K_{1}$ is the first modified Bessel function of the second kind (see [9]).

For the discrete wavelet case requiring strong additional conditions, the Meyer wavelets or the Gevrey wavelets constructed as in [10] belong to the Gelfand-Shilov spaces. As for the continuous wavelet transform requiring only the admissible condition, there are many possibilities to choose the analyzed wavelet. Boundedness results in a generalized Sobolev space, Besov space and Lizorkin-Triebel space are given in [3]. As for $\psi\in{\mathcal {S}}^{\mu}_{\nu}(\mathbf{R})$ and $\psi\in {\mathcal {S}}^{\mu,+}_{\nu}(\mathbf{R})$, [7] and [11] show the continuity properties of wavelet transforms by preparing spaces of functions in *a* and *b*, respectively. In this paper, we shall pay careful attention also to the parameter *h* as the radius of convergence in the analytic class and attempt to find a further detailed estimate with *h*. So, our purpose is to show the continuity properties in (strong) topologies of Banach Gelfand-Shilov spaces with the use of a vanishing moment condition and to give concrete examples which can indicate the optimality in Section 4.

## Results

To state our results, we also introduce the following lemma.

### Lemma 2.1

*There exist* $C>0$ *and* $h_{0}>0$ *such that*

$$\bigl\Vert e^{h_{0} \vert x \vert ^{1 /\nu}}f \bigr\Vert _{L^{\infty}(\mathbf{R}) }+ \bigl\Vert e^{h_{0} \vert \xi \vert ^{1 /\mu}}\hat{f} \bigr\Vert _{L^{\infty}(\mathbf{R}) } \leq C $$

*if and only if* $f \in{\mathcal {S}}^{\mu}_{\nu,h}(\mathbf{R}) $.

For the proof refer to [6, 12], etc. Taking Lemma 2.1 into account, we denote another Banach Gelfand-Shilov space combining with the infinite vanishing moment condition $\vert \hat{f} (\xi) \vert \leq C e^{-h \vert \xi \vert ^{-1 /\delta}}$,

$$S^{\mu,\delta}_{\nu, h}(\mathbf{R}) = \bigl\{ f \in{\mathcal {S}} ; \bigl\Vert e^{h \vert x \vert ^{1 /\nu}}f \bigr\Vert _{L^{\infty}(\mathbf{R}) }+ \bigl\Vert e^{h\max\{ \vert \xi \vert ^{1 /\mu}, \vert \xi \vert ^{-1 /\delta}\} }\hat{f} \bigr\Vert _{L^{\infty}(\mathbf{R}) } < \infty \bigr\} . $$

We remark that ${\mathcal {S}}^{\mu}_{\nu,h}(\mathbf{R}) $ corresponds to $S^{\mu,\delta}_{\nu, h}(\mathbf{R})$ with $\delta=\infty$, i.e.,

$$S^{\mu, \infty} _{\nu, h}(\mathbf{R}) = \bigl\{ f \in{\mathcal {S}} ; \bigl\Vert e^{h \vert x \vert ^{1 /\nu}}f \bigr\Vert _{L^{\infty}(\mathbf{R}) }+ \bigl\Vert e^{h \vert \xi \vert ^{1 /\mu}}\hat{f} \bigr\Vert _{L^{\infty}(\mathbf{R}) } < \infty \bigr\} . $$

### Remark 2.2

In particular, when $\hat{f} (\xi) $ is just equal to $e^{-h \vert \xi \vert ^{-1 /\delta}}$, it belongs to the Gevrey space of order $\delta+1$. So, *ν* can be taken as $\nu\geq\delta+1 $.

### Remark 2.3

We easily obtain $e^{h\max\{ \vert \xi \vert ^{1 /\mu}, \vert \xi \vert ^{-1 /\delta}\} }\leq e^{h( \vert \xi \vert ^{1 /\mu}+ \vert \xi \vert ^{-1 /\delta} ) }$. On the other hand, the weight can be estimated from below as

1 $$ e^{h\max\{ \vert \xi \vert ^{1 /\mu}, \vert \xi \vert ^{-1 /\delta}\} } \geq e^{h( \vert \xi \vert ^{1 /\mu}+ \vert \xi \vert ^{-1 /\delta}-1) }\geq c e^{h( \vert \xi \vert ^{1 /\mu }+ \vert \xi \vert ^{-1 /\delta} ) }. $$

Therefore, we find that $\Vert e^{h( \vert \xi \vert ^{1 /\mu}+ \vert \xi \vert ^{-1 /\delta})}\hat{f} \Vert _{L^{\infty}(\mathbf{R}) } \sim \Vert e^{h\max\{ \vert \xi \vert ^{1 /\mu}, \vert \xi \vert ^{-1 /\delta}\} }\hat{f} \Vert _{L^{\infty}(\mathbf{R}) } $.

Then we prove the following.

### Theorem 2.4

*Let* *μ*, *ν*, *h*, $h'$ *and* *δ* *be positive constants such that* $\mu+\nu\geq1$, $h'< h$. *Define that* $d(\lambda)= \lambda(\lambda-1)^{-1+1/\lambda} $. *Then*, *for the wavelet transform* $W_{\psi}$ *with the wavelet* $\psi\in S^{\mu,\delta}_{\nu, h}(\mathbf{R}) $, *the following estimates hold*:

- *for* $f\in S^{\infty, \infty} _{\nu, h}(\mathbf{R})$
  $$\begin{aligned}& (\mathrm{i}) \quad \biggl\Vert \frac{ e^{h \vert b/(a+1) \vert ^{1 /\nu}}}{ a^{1/2}+1 } W_{\psi}f \biggr\Vert _{L^{\infty}(\mathbf{R}_{+} \times \mathbf{R}) } \leq C \bigl\Vert e^{h \vert x \vert ^{1 /\nu}}f \bigr\Vert _{L^{\infty}(\mathbf{R}) }\quad \textit{if } \nu>1 , \\& (\mathrm{i})' \quad \bigl\Vert e^{ h' 2^{1-1/\nu} \vert b/(a+1) \vert ^{1 /\nu} } W_{\psi}f \bigr\Vert _{L^{\infty}(\mathbf{R}_{+} \times \mathbf{R}) } \leq C \bigl\Vert e^{h \vert x \vert ^{1 /\nu}}f \bigr\Vert _{L^{\infty}(\mathbf{R}) } \quad \textit{if } 0< \nu\leq1 , \end{aligned}$$
- *for* $f\in S^{\mu, \infty} _{\infty, h}(\mathbf{R})$
  $$\begin{aligned}& (\mathrm{ii})\quad \biggl\Vert \frac{a^{1/2}e^{h d(\delta/\mu+1)^{1/\mu} a^{-1/(\mu+\delta)} }}{a+1} W_{\psi}f \biggr\Vert _{L^{\infty}(\mathbf{R}_{+} \times \mathbf{R}) } \leq C \bigl\Vert e^{h \vert \xi \vert ^{1 /\mu}}\hat{f} \bigr\Vert _{L^{\infty}(\mathbf{R}) } \quad \textit{if } \mu>1 , \\& (\mathrm{ii})'\quad \bigl\Vert a^{-1/2} e^{ h' 2^{1-1/\mu} d(\delta/\mu+1)^{1/\mu } a^{-1 /(\mu+\delta)} } W_{\psi}f \bigr\Vert _{L^{\infty}(\mathbf{R}_{+} \times \mathbf{R}) } \leq C \bigl\Vert e^{h \vert \xi \vert ^{1 /\mu}}\hat{f} \bigr\Vert _{L^{\infty}(\mathbf{R}) }\\& \hphantom{(\mathrm{ii})'\quad }\quad \textit{if } 0< \mu\leq 1 , \end{aligned}$$
- *for* $f\in S^{\mu,\delta} _{\infty, h}(\mathbf{R})$
  $$\begin{aligned}& (\mathrm{iii})\quad \biggl\Vert \frac{a^{1/2} e^{h d(\delta/\mu+1)^{1/\mu} ( \max \{ a , a^{-1}\})^{1/(\mu+\delta) } }}{a+1} W_{\psi}f \biggr\Vert _{L^{\infty}(\mathbf{R}_{+} \times \mathbf{R}) } \\& \hphantom{(\mathrm{iii})\quad }\quad \leq C \bigl\Vert e^{h\max\{ \vert \xi \vert ^{1 /\mu}, \vert \xi \vert ^{-1 /\delta}\} } \hat{f} \bigr\Vert _{L^{\infty}(\mathbf{R}) } \quad \textit{if } \mu>1 , \\& (\mathrm{iii})' \quad \bigl\Vert a^{-1/2} e^{h' 2^{1-1/\mu} d(\delta/\mu +1)^{1/\mu} (\max\{ a , a^{-1}\})^{1/(\mu+\delta) } }W_{\psi}f \bigr\Vert _{L^{\infty}(\mathbf{R}_{+} \times \mathbf{R}) } \\& \hphantom{(\mathrm{iii})' \quad}\quad \leq C \bigl\Vert e^{h\max\{ \vert \xi \vert ^{1 /\mu}, \vert \xi \vert ^{-1 /\delta}\} } \hat{f} \bigr\Vert _{L^{\infty}(\mathbf{R}) } \quad \textit{if } 0< \mu\leq 1 . \end{aligned}$$

### Remark 2.5

We find that $d(\lambda)$ is strictly greater than 1 for $\lambda >1$ since $d'(\lambda)=-(\lambda-1)^{-1+1/\lambda}\lambda^{-1}\log(\lambda -1)$, and $d(\lambda)$ has the maximum at the point $\lambda=2$ and $\lim_{\lambda\rightarrow1+ }d(\lambda)=\lim_{\lambda\rightarrow\infty }d(\lambda)=1$.

### Remark 2.6

This work is motivated by [7] where *f* and *ψ* are allowed to take each different value of parameters *ν*, *μ* and have infinite vanishing moments, more precisely vanishing moments of arbitrary polynomial order. Therefore, we have restricted ourselves to the case of *f* and *ψ* under the common parameters *ν*, *μ*, and have derived the above estimates with *δ* (concerning vanishing moments of sub-exponential order). For instance, $\Vert e^{h ( \vert b \vert ^{1/(2\nu+\mu-1)} +(\max\{ a , a^{-1}\})^{1/(\nu+\mu-1) }) }W_{\psi}f \Vert _{L^{\infty}} $ is estimated by [7] with $\rho_{1}=s=\mu$, $\rho_{2}=\nu$, $\tau_{1}=\tau_{2} =\nu+ \mu -1$ and $t=2\nu+\mu-1$ (in the case of *f* and *ψ* under the common parameters *ν* and *μ*). If one considers small $a>0$ and takes $\nu=\delta+1$ (see Remark 2.2), a similar estimate as (ii) holds since $(\max\{ a , a^{-1}\})^{1/( \nu+\mu-1 ) }(\max\{ a , a^{-1}\})^{1/( \mu+\delta) } \sim a^{-1/( \mu+\delta) }$. Thanks to the additional condition of sub-exponential order, (i) for small $a>0$ can become better since $\mu+\nu\geq1$ and $\vert b \vert ^{1/(2\nu+\mu-1)}= \vert b \vert ^{1/(\nu+(\mu+\nu-1))}\leq \vert b \vert ^{1/\nu}\sim \vert b/ (a+1) \vert ^{1/\nu}$.

Considering the study of the continuity properties in [3, 7] and [11], we introduce spaces of functions in *a* and *b* which correspond to the Gelfand-Shilov spaces of functions in *x* and *ξ* since $a\sim 1/ \vert \xi \vert $ and $b \sim x$ after wavelet transforms. Therefore, we shall define the following weighted $L^{\infty}(\mathbf {R}_{+} \times \mathbf{R})$ space which is a subspace of $L^{2}(\mathbf {R}_{+} \times \mathbf{R})$ as far as *h* is positive:

$$V^{ \mu,\delta} _{\nu, h}(\mathbf{R}_{+} \times\mathbf{R}) = \bigl\{ F \in L^{2}(\mathbf{R}_{+} \times\mathbf{R}) ; \bigl\Vert e^{h \max\{ \vert b/(a+1) \vert ^{1 /\nu}, a^{1 /\mu}, a^{-1 /\delta} \} } F \bigr\Vert _{L^{\infty}(\mathbf{R}_{+} \times \mathbf{R}) } < \infty \bigr\} . $$

We remark that if $\mu=\infty$,

$$V^{\infty,\delta}_{\nu, h}(\mathbf{R}_{+} \times\mathbf{R}) = \bigl\{ F \in L^{2}(\mathbf{R}_{+} \times\mathbf{R}) ; \bigl\Vert e^{h \max\{ \vert b/(a+1) \vert ^{1 /\nu}, a^{-1 /\delta}\} } F \bigr\Vert _{L^{\infty}(\mathbf{R}_{+} \times \mathbf{R}) } < \infty \bigr\} . $$

By (i) and (ii), we have

$$\begin{aligned}& \biggl\Vert \biggl\{ \frac{ e^{h \vert b/(a+1) \vert ^{1 /\nu }}}{ a^{1/2} +1} + \frac{a^{1/2} e^{h d(\delta/\mu+1)^{1/\mu} a^{-1 /(\mu+\delta)}}}{a+1} \biggr\} W_{\psi}f \biggr\Vert _{L^{\infty}(\mathbf{R}_{+} \times \mathbf{R}) } \\& \quad \leq C \bigl( \bigl\Vert e^{h \vert x \vert ^{1 /\nu}}f \bigr\Vert _{L^{\infty}(\mathbf {R}) }+ \bigl\Vert e^{h \vert \xi \vert ^{1 /\mu}} \hat{f} \bigr\Vert _{L^{\infty}(\mathbf{R}) } \bigr) . \end{aligned}$$

The weight function can be estimated from below as

$$\frac{ e^{h \vert b/(a+1) \vert ^{1 /\nu}}}{ a^{1/2} +1} + \frac {a^{1/2} e^{h d(\delta/\mu+1)^{1/\mu} a^{-1 /(\mu+\delta)}}}{a+1} \geq c e^{h \max\{ \vert b/(a+1) \vert ^{1 /\nu}, a^{-1 /(\mu +\delta) }\} } , $$

here we used Remark 2.5 also to eliminate the term $a^{1/2} $. Therefore, by Theorem 2.4, we can also get the following continuity properties.

### Corollary 2.7

*Let* *μ*, *ν*, *h* *and* *δ* *be constants such that* $\mu>1$, $\nu >1$, $h>0$ *and* $\delta>0$. *Then*, *for the wavelet* $\psi\in S^{\mu,\delta}_{\nu, h}(\mathbf {R}) $, *the wavelet transform* $S^{\mu,\infty}_{\nu, h}(\mathbf{R}) \ni f \mapsto W_{\psi}f \in V^{\infty, \mu+\delta}_{\nu, h }(\mathbf{R}_{+} \times \mathbf{R})$ *is continuous*. *In particular*, *when* *f* *also satisfies the infinite vanishing moment condition*, *the wavelet transform* $S^{\mu,\delta}_{\infty, h}(\mathbf{R}) \ni f \mapsto W_{\psi}f \in V^{ \mu+\delta, \mu+ \delta}_{\infty, h }(\mathbf{R}_{+} \times \mathbf{R}) $ *is continuous*.

In Section 4 we shall discuss the optimality of our boundedness results in Gelfand-Shilov spaces.

## Proof of Theorem 2.4

At first, we introduce the following lemma.

### Lemma 3.1

*It holds that for* $\alpha, \beta\geq0$

$$\alpha^{1/\theta} +\beta^{1/\theta} \geq \textstyle\begin{cases} 2^{1-1/\theta} ( \alpha + \beta)^{1/\theta} & \textit{if } 0< \theta\leq1 , \\ ( \alpha+ \beta)^{1/\theta} + (2 -2^{1/\theta})\min\{ \alpha^{1/\theta}, \beta^{1/\theta} \} &\textit{if } \theta> 1 . \end{cases} $$

### Remark 3.2

The latter inequality is given in [13] and [14], which also shows multiplication algebras for the Gevrey-modulation spaces.

### Proof of Lemma 3.1

We shall suppose that $\alpha\geq\beta> 0$ since the proof is trivial when $\alpha=0$ or $\beta=0$. Putting $\gamma :=\alpha/\beta$ ( ≥1), we may show

$$\gamma^{1/\theta} +1 \geq \textstyle\begin{cases} 2^{1-1/\theta} ( \gamma+ 1)^{1/\theta} &\text{if } 0< \theta\leq1 , \\ ( \gamma+ 1 )^{1/\theta} + 2 -2^{1/\theta} &\text{if } \theta> 1 . \end{cases} $$

This follows from

$$\min_{\gamma \geq1} \biggl\{ \frac{\gamma^{1/\theta} +1}{ ( \gamma+ 1)^{1/\theta} } \biggr\} = 2^{1-1/\theta} \quad \text{if } 0< \theta\leq1 , $$

and

$$\min_{\gamma \geq1} \bigl\{ \gamma^{1/\theta} +1 - ( \gamma+ 1)^{1/\theta} \bigr\} =2 -2^{1/\theta} \quad \text{if } \theta> 1. $$

 □

In the proofs of theorems, $\Vert \cdot \Vert $ denotes the $L^{\infty}$ norm on **R** or $\mathbf{R}_{+} \times \mathbf{R}$. We shall consider the following cases.

• Case of $\nu>1$ and $a\geq1$) From the definition of the wavelet transform we get

$$\bigl\vert e^{h \vert b/ \max\{1,a \} \vert ^{1 /\nu}}W_{\psi}f (a,b) \bigr\vert \leq \frac{C \Vert e^{h \vert x \vert ^{1 /\nu}}f \Vert }{a^{1/2}} \int_{\mathbf{R}}e^{ -h \{ \vert x \vert ^{1/\nu} + \vert (x-b)/a \vert ^{1/\nu} - \vert b/ \max\{1,a\} \vert ^{1 /\nu}\} } \,dx. $$

Lemma 3.1 with $\alpha= \vert b/a -x/a \vert $, $\beta= \vert x/a \vert $ gives

$$\begin{aligned}& \vert x \vert ^{1/\nu} + \biggl\vert \frac{x-b}{a} \biggr\vert ^{1/\nu} - \biggl\vert \frac{b}{\max\{1,a\} } \biggr\vert ^{1/\nu} \\& \quad = \biggl\vert \frac{b}{a} -\frac{x}{a} \biggr\vert ^{1/\nu} + \biggl\vert \frac{x}{a} \biggr\vert ^{1/\nu} - \biggl\vert \frac{b}{a} \biggr\vert ^{1/\nu} + \vert x \vert ^{1/\nu} \biggl\{ 1- \biggl\vert \frac{1}{a} \biggr\vert ^{1/\nu} \biggr\} \\& \quad \geq \biggl( \biggl\vert \frac{b}{a} - \frac{x}{a} \biggr\vert + \biggl\vert \frac{x}{a} \biggr\vert \biggr)^{1/\nu} + \bigl(2 -2^{1/\nu} \bigr)\min \biggl\{ \biggl\vert \frac{b}{a} - \frac{x}{a} \biggr\vert ^{1/\nu} , \biggl\vert \frac{x}{a} \biggr\vert ^{1/\nu} \biggr\} - \biggl\vert \frac{b}{a} \biggr\vert ^{1/\nu} \\& \quad\quad{} + \vert x \vert ^{1/\nu} \biggl\{ 1- \biggl\vert \frac{1}{a} \biggr\vert ^{1/\nu} \biggr\} \\& \quad \geq { \bigl(2 -2^{1/\nu} \bigr)\min \biggl\{ \biggl\vert \frac{b}{a} -\frac{x}{a} \biggr\vert ^{1/\nu} , \biggl\vert \frac{x}{a} \biggr\vert ^{1/\nu} \biggr\} + \vert x \vert ^{1/\nu} \biggl\{ 1- \biggl\vert \frac{1}{a} \biggr\vert ^{1/\nu} \biggr\} ,} \end{aligned}$$

here we used $( \vert b/a -x/a \vert + \vert x/a \vert )^{1/\nu} \geq \vert b/a \vert ^{1/\nu} $. Therefore, putting $D:= \{ x \in\mathbf{R}; \vert b/a -x/a \vert < \vert x/a \vert \}$, we have

$$\begin{aligned} \bigl\vert e^{h \vert b/ \max\{1,a\} \vert ^{1 /\nu}}W_{\psi}f (a,b) \bigr\vert \leq& \frac{C \Vert e^{h \vert x \vert ^{1 /\nu}}f \Vert }{a^{1/2}} \int_{D} e^{-h(2 -2^{1/\nu}) \vert b/a -x/a \vert ^{1/\nu} } \,dx \\ & {}+\frac{C \Vert e^{h \vert x \vert ^{1 /\nu}}f \Vert }{a^{1/2}} \int_{\mathbf{R}\backslash D}e^{-h(2 -2^{1/\nu}) \vert x/a \vert ^{1/\nu} } \,dx \\ \leq& 2 Ca^{1/2} \bigl\Vert e^{h \vert x \vert ^{1 /\nu}}f \bigr\Vert \int_{\mathbf{R}} e^{-h(2 -2^{1/\nu}) \vert x \vert ^{1/\nu} } \,dx \\ \leq& C a^{1/2} \bigl\Vert e^{h \vert x \vert ^{1 /\nu}}f \bigr\Vert . \end{aligned}$$

• Case of $\nu>1$ and $0 < a< 1$) Lemma 3.1 with $\alpha = \vert b-x \vert $, $\beta= \vert x \vert $ gives

$$\begin{aligned}& \vert x \vert ^{1/\nu} + \biggl\vert \frac{x-b}{a} \biggr\vert ^{1/\nu} - \biggl\vert \frac{b}{\max\{1,a\} } \biggr\vert ^{1/\nu} \\& \quad = \vert b-x \vert ^{1/\nu}+ \vert x \vert ^{1/\nu} - \vert b \vert ^{1/\nu} + \vert b-x \vert ^{1/\nu} \biggl\{ \biggl\vert \frac{1}{a} \biggr\vert ^{1/\nu} -1 \biggr\} \\& \quad \geq { \bigl( \vert b-x \vert + \vert x \vert \bigr)^{1/\nu} + \bigl(2 -2^{1/\nu} \bigr)\min \bigl\{ \vert b -x \vert ^{1/\nu} , \vert x \vert ^{1/\nu} \bigr\} - \vert b \vert ^{1/\nu} + \vert b-x \vert ^{1/\nu} \biggl\{ \biggl\vert \frac{1}{a} \biggr\vert ^{1/\nu} -1 \biggr\} } \\& \quad \geq { \bigl(2 -2^{1/\nu} \bigr)\min \bigl\{ \vert b -x \vert ^{1/\nu} , \vert x \vert ^{1/\nu} \bigr\} + \vert b-x \vert ^{1/\nu} \biggl\{ \biggl\vert \frac{1}{a} \biggr\vert ^{1/\nu} -1 \biggr\} ,} \end{aligned}$$

here we used $( \vert b-x \vert + \vert x \vert )^{1/\nu} \geq \vert b \vert ^{1/\nu} $. Therefore, putting

$$I:= \bigl\{ x \in\mathbf{R}; \vert b -x \vert < 1 \bigr\} , $$

we find that

$$\begin{aligned} \int_{I} e^{-h \vert b-x \vert ^{1/\nu} \{ \vert 1/a \vert ^{1/\nu} -1 \} } \,dx = & \int^{b+1}_{b-1} e^{-h \vert (b-x)/a \vert ^{1/\nu} \{ 1- a^{1/\nu} \} } \,dx \\ = & a \int^{ 1/a }_{- 1/a } e^{-h \vert x \vert ^{1/\nu} \{ 1- a^{1/\nu} \} } \,dx \leq M_{h ,\nu}a \end{aligned}$$

for $0< a<1$ and have

$$\begin{aligned}& \bigl\vert e^{h \vert b/ \max\{1,a\} \vert ^{1 /\nu}}W_{\psi}f (a,b) \bigr\vert \\& \quad \leq \frac{C \Vert e^{h \vert x \vert ^{1 /\nu}}f \Vert }{a^{1/2}} \int_{I} e^{-h \vert b-x \vert ^{1/\nu} \{ \vert 1/a \vert ^{1/\nu} -1 \} } \,dx \\& \quad\quad{} +\frac{C \Vert e^{h \vert x \vert ^{1 /\nu}}f \Vert }{a^{1/2}} \int_{\mathbf{R}\backslash I}e^{-h \{ (2 -2^{1/\nu})\min\{ \vert b -x \vert ^{1/\nu} , \vert x \vert ^{1/\nu}\} + \vert 1/a \vert ^{1/\nu} -1 \} } \,dx \\& \quad \leq { M_{h,\nu}a^{1/2} \bigl\Vert e^{h \vert x \vert ^{1 /\nu}}f \bigr\Vert } \\& \quad\quad{} + M_{h,\nu}' \bigl\Vert e^{h \vert x \vert ^{1 /\nu}}f \bigr\Vert \int_{\mathbf{R} }e^{-h (2 -2^{1/\nu})\min\{ \vert b -x \vert ^{1/\nu} , \vert x \vert ^{1/\nu}\} } \,dx \\& \quad \leq C \bigl\Vert e^{h \vert x \vert ^{1 /\nu}}f \bigr\Vert , \end{aligned}$$

here we used $\frac{1}{ a^{1/2}} e^{-h \{ \vert 1/a \vert ^{1/\nu}-1\} } \leq M_{h,\nu} ' $ for $0< a<1$.

Thus, since $\max\{ 1 ,a \} \leq a+1 $ and $\max\{ 1 ,a^{1/2}\} \sim a^{1/2} +1 $, it follows that

$$\biggl\Vert \frac{ e^{h \vert b/(a+1) \vert ^{1 /\nu}}}{ a^{1/2}+1 } W_{\psi}f \biggr\Vert \leq C \bigl\Vert e^{h \vert x \vert ^{1 /\nu}}f \bigr\Vert . $$

• Case of $0<\nu\leq1$ and $a\geq1$) For $h>h'>0$, we get

$$\begin{aligned}& \bigl\vert e^{ h' 2^{1-1/\nu} \vert b/\max\{1,a\} \vert ^{1 /\nu} } W_{\psi}f (a,b) \bigr\vert \\& \quad \leq \frac{C \Vert e^{h \vert x \vert ^{1 /\nu}}f \Vert }{a^{1/2}} \\& \quad \quad{} \times \int^{\infty}_{-\infty} e^{ -(h-h' )\{ \vert x \vert ^{1/\nu} + \vert (x-b)/a \vert ^{1/\nu} \} -h' \{ \vert x \vert ^{1/\nu} + \vert (x-b)/a \vert ^{1/\nu} - 2^{1-1/\nu} \vert b/\max\{1,a\} \vert ^{1 /\nu }\} } \,dx. \end{aligned}$$

Lemma 3.1 with $\alpha= \vert b/a -x/a \vert $, $\beta= \vert x/a \vert $ gives

$$\begin{aligned}& \vert x \vert ^{1/\nu} + \biggl\vert \frac{x-b}{a} \biggr\vert ^{1/\nu} - 2^{1-1/\nu} \biggl\vert \frac{b}{\max\{1,a\} } \biggr\vert ^{1/\nu} \\& \quad = \biggl\vert \frac{b}{a} -\frac{x}{a} \biggr\vert ^{1/\nu} + \biggl\vert \frac{x}{a} \biggr\vert ^{1/\nu} -2^{1-1/\nu} \biggl\vert \frac{b}{a} \biggr\vert ^{1/\nu} + \vert x \vert ^{1/\nu} \biggl\{ 1- \biggl\vert \frac{1}{a} \biggr\vert ^{1/\nu} \biggr\} \\& \quad \geq { 2^{1-1/\nu} \biggl( \biggl\vert \frac{b}{a} - \frac{x}{a} \biggr\vert + \biggl\vert \frac{x}{a} \biggr\vert \biggr)^{1/\nu} - 2^{1-1/\nu} \biggl\vert \frac{b}{a} \biggr\vert ^{1/\nu} + \vert x \vert ^{1/\nu} \biggl\{ 1- \biggl\vert \frac{1}{a} \biggr\vert ^{1/\nu} \biggr\} } \\& \quad \geq { \vert x \vert ^{1/\nu} \biggl\{ 1- \biggl\vert \frac{1}{a} \biggr\vert ^{1/\nu} \biggr\} .} \end{aligned}$$

Therefore, we have

$$\begin{aligned} \bigl\vert e^{ h' 2^{1-1/\nu} \vert b/\max\{1,a\} \vert ^{1 /\nu } } W_{\psi}f (a,b) \bigr\vert \leq& \frac{C \Vert e^{h \vert x \vert ^{1 /\nu}}f \Vert }{a^{1/2}} \int^{\infty}_{-\infty} e^{ -(h-h' )\{ \vert x \vert ^{1/\nu} + \vert (x-b)/a \vert ^{1/\nu} \} } \,dx \\ \leq& \frac{C \Vert e^{h \vert x \vert ^{1 /\nu}}f \Vert }{a^{1/2}} \int^{\infty}_{-\infty} e^{ -(h-h' ) \vert x \vert ^{1/\nu} } \,dx \\ \leq&{ C \bigl\Vert e^{h \vert x \vert ^{1 /\nu}}f \bigr\Vert . } \end{aligned}$$

• Case of $0<\nu\leq1$ and $0 < a< 1$) Lemma 3.1 with $\alpha= \vert b-x \vert $, $\beta= \vert x \vert $ gives

$$\begin{aligned}& \vert x \vert ^{1/\nu} + \biggl\vert \frac{x-b}{a} \biggr\vert ^{1/\nu} - 2^{1-1/\nu} \biggl\vert \frac{b}{\max\{1,a\} } \biggr\vert ^{1/\nu} \\ & \quad = \vert b-x \vert ^{1/\nu}+ \vert x \vert ^{1/\nu} - 2^{1-1/\nu} \vert b \vert ^{1/\nu} + \vert b-x \vert ^{1/\nu} \biggl\{ \biggl\vert \frac{1}{a} \biggr\vert ^{1/\nu} -1 \biggr\} \\ & \quad \geq { 2^{1-1/\nu} \bigl( \vert b-x \vert + \vert x \vert \bigr)^{1/\nu} - 2^{1-1/\nu} \vert b \vert ^{1/\nu} + \vert b-x \vert ^{1/\nu} \biggl\{ \biggl\vert \frac{1}{a} \biggr\vert ^{1/\nu} -1 \biggr\} } \\ & \quad \geq { \vert b-x \vert ^{1/\nu} \biggl\{ \biggl\vert \frac{1}{a} \biggr\vert ^{1/\nu} -1 \biggr\} . } \end{aligned}$$

Therefore, we have

$$\begin{aligned} \bigl\vert e^{ h' 2^{1-1/\nu} \vert b/\max\{1,a\} \vert ^{1 /\nu } } W_{\psi}f (a,b) \bigr\vert \leq& \frac{C \Vert e^{h \vert x \vert ^{1 /\nu}}f \Vert }{a^{1/2}} \int^{\infty}_{-\infty} e^{ -(h-h' )\{ \vert x \vert ^{1/\nu} + \vert (x-b)/a \vert ^{1/\nu} \} } \,dx \\ \leq& \frac{C \Vert e^{h \vert x \vert ^{1 /\nu}}f \Vert }{a^{1/2}} \int^{\infty}_{-\infty} e^{ -(h-h' ) \vert (x-b)/a \vert ^{1/\nu} } \,dx \\ \leq&{ C \bigl\Vert e^{h \vert x \vert ^{1 /\nu}}f \bigr\Vert . } \end{aligned}$$

Thus, since $\max\{ 1 ,a \} \leq a+1 $, it follows that

$$\bigl\Vert e^{ h' 2^{1-1/\nu} \vert b/(a+1) \vert ^{1 /\nu} } W_{\psi}f \bigr\Vert \leq C \bigl\Vert e^{h \vert x \vert ^{1 /\nu}}f \bigr\Vert . $$

• Case of $\mu>1$) Let $\mu' := \mu/(\mu+\delta) $. By Parseval’s theorem, the wavelet transform can be rewritten as

2 $$ W_{\psi}f (a,b)=\sqrt{ \frac{a}{ C_{\psi}}} \int_{\mathbf{R}}\hat{f}(\xi) \overline{e^{-ib\xi}\hat{\psi}(a \xi ) }\,d\xi. $$

Since

$$e^{h\max\{ \vert a\xi \vert ^{1 /\mu}, \vert a\xi \vert ^{-1 /\delta}\} } \geq e^{h( \vert a\xi \vert ^{1 /\mu}+ \vert a\xi \vert ^{-1 /\delta}-1) }\geq c e^{h( \vert a\xi \vert ^{1 /\mu}+ \vert a\xi \vert ^{-1 /\delta} ) } $$

similarly as (1), we see that

$$\bigl\vert e^{-ib\xi}\hat{\psi}(a\xi) \bigr\vert \leq Ce^{-h( \vert a\xi \vert ^{1 /\mu}+ \vert a\xi \vert ^{-1 /\delta} ) }. $$

Hence, we get

$$\begin{aligned}& \bigl\vert e^{h\{ d(\delta/\mu+1) (1+1/a)^{\mu'}\}^{1/\mu} }W_{\psi}f (a,b) \bigr\vert \\ & \quad \leq Ca^{1/2} \bigl\Vert e^{h \vert \xi \vert ^{1 /\mu}}\hat{f} \bigr\Vert \int^{\infty}_{-\infty} e^{ -h\{ \vert \xi \vert ^{1/\mu} + \vert a\xi \vert ^{1 /\mu } + \vert a\xi \vert ^{-1 /\delta} - \{ d(\delta/\mu+1) (1+1/a)^{\mu'}\}^{1/\mu} \} } \,d\xi. \end{aligned}$$

Lemma 3.1 with $\alpha= \vert \xi \vert $, $\beta= \vert a\xi \vert + \vert a\xi \vert ^{-\mu/\delta}$ gives

$$\begin{aligned}& \vert \xi \vert ^{1/\mu} + \vert a\xi \vert ^{1 /\mu} + \frac{1}{ \vert a\xi \vert ^{1 /\delta} }- \biggl\{ d(\delta/\mu +1) \biggl(1+\frac{1}{a} \biggr)^{\mu'} \biggr\} ^{1/\mu} \\ & \quad \geq \vert \xi \vert ^{1/\mu} + \biggl( \vert a\xi \vert + \frac{1}{ \vert a\xi \vert ^{\mu/\delta} } \biggr)^{1 /\mu} - \biggl\{ d(\delta/\mu+1) \biggl(1+ \frac{1}{a} \biggr)^{\mu'} \biggr\} ^{1/\mu} \\& \quad \geq { \biggl( \vert \xi \vert + \vert a\xi \vert + \frac{1}{ \vert a\xi \vert ^{\mu/\delta}} \biggr)^{1 /\mu} + \bigl(2 -2^{1/\mu} \bigr)\min \biggl\{ \vert \xi \vert ^{1/\mu} , \biggl( \vert a\xi \vert + \frac{1}{ \vert a\xi \vert ^{\mu/\delta} } \biggr)^{1 /\mu} \biggr\} } \\& \quad\quad{} - \biggl\{ d(\delta/\mu+1) \biggl(1+\frac{1}{a} \biggr)^{\mu'} \biggr\} ^{1/\mu} \\& \quad \geq \bigl(2 -2^{1/\mu} \bigr)\min \biggl\{ \vert \xi \vert ^{1/\mu} , \biggl( \vert a\xi \vert +\frac{1}{ \vert a\xi \vert ^{\mu/\delta}} \biggr)^{1 /\mu} \biggr\} , \end{aligned}$$

here we used

$$\min_{\xi\in\mathbf{R}} \biggl( \vert \xi \vert + \vert a\xi \vert + \frac{1}{ \vert a\xi \vert ^{\mu/\delta} } \biggr) = d \biggl(\frac{\delta}{\mu}+1 \biggr) \biggl(1+ \frac{1}{a} \biggr)^{\mu'} . $$

Therefore, putting

$$D:= \bigl\{ \xi\in\mathbf{R}; \vert \xi \vert ^{1/\mu} < \bigl( \vert a\xi \vert + \vert a\xi \vert ^{-\mu/\delta} \bigr)^{1 /\mu} \bigr\} , $$

we have

$$\begin{aligned}& \bigl\vert e^{h\{ d(\delta/\mu+1) (1+1/a)^{\mu'} \}^{1/\mu} }W_{\psi}f (a,b) \bigr\vert \\& \quad \leq Ca^{1/2} \bigl\Vert e^{h \vert \xi \vert ^{1 /\mu }}\hat{f} \bigr\Vert \int_{D} e^{-h(2 -2^{1/\mu}) \vert \xi \vert ^{1/\mu} } \,d\xi \\& \quad\quad{} + Ca^{1/2} \bigl\Vert e^{h \vert \xi \vert ^{1 /\mu}}\hat{f} \bigr\Vert \int_{\mathbf{R}\backslash D}e^{-h(2 -2^{1/\mu}) ( \vert a\xi \vert + \vert a\xi \vert ^{-\mu/\delta} )^{1 /\mu} } \,d\xi \\& \quad \leq \textstyle\begin{cases} C a^{1/2} \Vert e^{h \vert \xi \vert ^{1 /\mu}}\hat{f} \Vert \int_{\mathbf{R}} e^{-h(2 -2^{1/\mu}) \vert \xi \vert ^{1/\mu} } \,d\xi& \text{if } a\geq1 , \\ \frac{ C \Vert e^{h \vert \xi \vert ^{1 /\mu}}\hat{f} \Vert }{a^{1/2}}\int_{\mathbf{R}} e^{-h(2 -2^{1/\mu}) \vert \xi \vert ^{1/\mu} } \,d\xi &\text{if } 0 < a< 1 \end{cases}\displaystyle \\& \quad \leq C \max \bigl\{ a^{1/2},a^{-1/2} \bigr\} \bigl\Vert e^{h \vert \xi \vert ^{1 /\mu}}\hat{f} \bigr\Vert . \end{aligned}$$

Thus, since

$$e^{h \{ d(\delta/\mu+1) (1+1/a)^{\mu'} \}^{1/\mu} }\geq e^{h\{ d(\delta /\mu+1) a^{-\mu'} \}^{1/\mu} } = e^{h d(\delta/\mu+1)^{1/\mu} a^{-1/(\mu +\delta)} } $$

and $\max\{ a^{1/2} ,a^{-1/2}\}= a^{-1/2}\max\{ a,1 \}\sim a^{-1/2}(1+a)$, it follows that

$$\biggl\Vert \frac{a^{1/2} e^{h d(\delta/\mu+1)^{1/\mu} a^{-1/(\mu +\delta)} }}{1+a} W_{\psi}f \biggr\Vert \leq C \bigl\Vert e^{h \vert \xi \vert ^{1 /\mu}}\hat{f} \bigr\Vert . $$

• Case of $\mu>1$ with the condition $\vert \hat{f} (\xi) \vert \leq C e^{-h \vert \xi \vert ^{-1 /\delta}}$) Let $\delta' := \delta/(\mu+\delta) $. By (2) we get

$$\begin{aligned}& \bigl\vert e^{h\{ d(\delta/\mu+1) (1+a )^{\mu'} (1+1/a^{\mu/\delta})^{\delta'}\}^{1/\mu} }W_{\psi}f (a,b) \bigr\vert \\& \quad \leq Ca^{1/2} \bigl\Vert e^{h ( \vert \xi \vert ^{1 /\mu}+ \vert \xi \vert ^{-1 /\delta})}\hat{f} \bigr\Vert \\& \quad\quad{} \times \int^{\infty}_{-\infty} e^{ -h\{ \vert \xi \vert ^{1/\mu} + \vert \xi \vert ^{-1 /\delta}+ \vert a\xi \vert ^{1 /\mu} + \vert a\xi \vert ^{-1 /\delta} - \{ d(\delta/\mu+1) (1+a )^{\mu'} (1+1/a^{\mu/\delta})^{\delta'}\}^{1/\mu} } \,d\xi. \end{aligned}$$

Lemma 3.1 with $\alpha= \vert \xi \vert + \vert \xi \vert ^{-\mu/\delta}$, $\beta= \vert a\xi \vert + \vert a\xi \vert ^{-\mu/\delta}$ gives

$$\begin{aligned}& \vert \xi \vert ^{1/\mu} +\frac{1}{ \vert \xi \vert ^{1 /\delta} }+ \vert a\xi \vert ^{1 /\mu} +\frac{1}{ \vert a\xi \vert ^{1 /\delta} }- \biggl\{ d(\delta/\mu+1) (1+ a )^{\mu'} \biggl(1+\frac{1}{a^{\mu/\delta} } \biggr)^{\delta'} \biggr\} ^{1/\mu} \\& \quad \geq \biggl( \vert \xi \vert +\frac{1}{ \vert \xi \vert ^{\mu/\delta} } \biggr)^{1 /\mu} + \biggl( \vert a\xi \vert +\frac{1}{ \vert a\xi \vert ^{\mu /\delta} } \biggr)^{1 /\mu} - \biggl\{ d( \delta/\mu+1) (1+ a )^{\mu'} \biggl(1+\frac{1}{a^{\mu/\delta} } \biggr)^{\delta'} \biggr\} ^{1/\mu} \\& \quad \geq \biggl( \vert \xi \vert +\frac{1}{ \vert \xi \vert ^{\mu/\delta} } + \vert a\xi \vert + \frac{1}{ \vert a\xi \vert ^{\mu/\delta}} \biggr)^{1 /\mu} \\& \quad\quad{} + \bigl(2 -2^{1/\mu} \bigr)\min \biggl\{ \biggl( \vert \xi \vert + \frac{1}{ \vert \xi \vert ^{\mu/\delta}} \biggr)^{1 /\mu} , \biggl( \vert a\xi \vert + \frac{1}{ \vert a\xi \vert ^{\mu/\delta} } \biggr)^{1 /\mu} \biggr\} \\& \quad\quad{} - \biggl\{ d(\delta/\mu+1) (1+ a )^{\mu'} \biggl(1+ \frac{1}{a^{\mu/\delta} } \biggr)^{\delta'} \biggr\} ^{1/\mu} \\& \quad \geq { \bigl(2 -2^{1/\mu} \bigr)\min \biggl\{ \biggl( \vert \xi \vert + \frac{1}{ \vert \xi \vert ^{\mu/\delta}} \biggr)^{1 /\mu} , \biggl( \vert a \xi \vert + \frac{1}{ \vert a\xi \vert ^{\mu/\delta}} \biggr)^{1 /\mu} \biggr\} , } \end{aligned}$$

here we used

$$\min_{\xi\in\mathbf{R}} \biggl( \vert \xi \vert +\frac{1}{ \vert \xi \vert ^{\mu/\delta}}+ \vert a\xi \vert +\frac{1}{ \vert a\xi \vert ^{\mu/\delta} } \biggr) = d \biggl(\frac{\delta}{\mu}+1 \biggr) (1+ a )^{\mu'} \biggl(1+\frac{1}{a^{\mu/\delta} } \biggr)^{\delta'}. $$

Therefore, putting

$$D:= \bigl\{ \xi\in\mathbf{R}; \bigl( \vert \xi \vert + \vert \xi \vert ^{-\mu/\delta} \bigr)^{1 /\mu} < \bigl( \vert a\xi \vert + \vert a \xi \vert ^{-\mu/\delta} \bigr)^{1 /\mu} \bigr\} , $$

we have

$$\begin{aligned}& \bigl\vert e^{h\{ d(\delta/\mu+1) (1+a )^{\mu'} (1+1/a^{\mu/\delta})^{\delta'}\}^{1/\mu}}W_{\psi}f (a,b) \bigr\vert \\& \quad \leq Ca^{1/2} \bigl\Vert e^{h ( \vert \xi \vert ^{1 /\mu}+ \vert \xi \vert ^{-1 /\delta})}\hat{f} \bigr\Vert \int_{D} e^{-h(2 -2^{1/\mu}) ( \vert \xi \vert + \vert \xi \vert ^{-\mu/\delta} )^{1/\mu} } \,d\xi \\& \quad\quad{} + Ca^{1/2} \bigl\Vert e^{h ( \vert \xi \vert ^{1 /\mu}+ \vert \xi \vert ^{-1 /\delta})}\hat{f} \bigr\Vert \int_{\mathbf{R}\backslash D}e^{-h(2 -2^{1/\mu}) ( \vert a\xi \vert + \vert a\xi \vert ^{-\mu/\delta} )^{1 /\mu} } \,d\xi \\& \quad \leq \textstyle\begin{cases} C a^{1/2} \Vert e^{h ( \vert \xi \vert ^{1 /\mu}+ \vert \xi \vert ^{-1 /\delta})}\hat{f} \Vert \int_{\mathbf{R}} e^{-h(2 -2^{1/\nu}) \vert \xi \vert ^{1/\mu} } \,d\xi& \text{if } a\geq1 , \\ \frac{ C \Vert e^{h ( \vert \xi \vert ^{1 /\mu}+ \vert \xi \vert ^{-1 /\delta})}\hat{f} \Vert }{a^{1/2}}\int_{\mathbf{R}} e^{-h(2 -2^{1/\nu}) \vert \xi \vert ^{1/\mu} } \,d\xi & \text{if } 0 < a< 1 \end{cases}\displaystyle \\& \quad \leq C\max \bigl\{ a^{1/2},a^{-1/2} \bigr\} \bigl\Vert e^{h ( \vert \xi \vert ^{1 /\mu}+ \vert \xi \vert ^{-1 /\delta})}\hat{f} \bigr\Vert . \end{aligned}$$

Thus, since

3 $$ (1+a )^{\mu'} \biggl(1+\frac{1}{a^{\mu/\delta}} \biggr)^{\delta'} \geq \biggl( \max \biggl\{ a ,\frac{1}{a} \biggr\} \biggr)^{\mu'}, $$

and

$$e^{h \{ d(\delta/\mu+1) (1+a )^{\mu'} (1+1/a^{\mu/\delta})^{\delta'}\}^{1/\mu}}\geq e^{hd(\delta/\mu +1)^{1/\mu} ( \max\{ a , a^{- 1}\}) ^{1/(\mu+ \delta) } } , $$

it follows that

$$\biggl\Vert \frac{a^{1/2} e^{h d(\delta/\mu+1)^{1/\mu} ( \max\{ a , a^{-1}\}) ^{1/(\mu+\delta) } }}{1+a} W_{\psi}f \Vert \leq C \Vert e^{h ( \vert \xi \vert ^{1 /\mu}+ \vert \xi \vert ^{-1 /\delta})}\hat{f} \biggr\Vert . $$

• Case of $0<\mu\leq1$) Let $k = \max\{ 1 /\mu-1, 1\} -1/\delta$. For $h>h'>0$, we get

$$\begin{aligned}& \bigl\vert e^{ h' 2^{1-1/\mu} \{ d(\delta/\mu+1) (1+1/a )^{\mu '} \}^{1/\mu} }W_{\psi}f (a,b) \bigr\vert \\& \quad \leq Ca^{1/2} \bigl\Vert e^{h \vert \xi \vert ^{1 /\mu}}\hat{f} \bigr\Vert \\& \quad \quad{} \times \int^{\infty}_{-\infty} e^{ -(h-h')\{ \vert \xi \vert ^{1/\mu} + \vert a\xi \vert ^{1 /\mu} + \vert a\xi \vert ^{-1 /\delta}\}} \\& \quad \quad{} \times e^{-h' \{ \vert \xi \vert ^{1/\mu} + \vert a\xi \vert ^{1 /\mu} + \vert a\xi \vert ^{-1 /\delta} - 2^{1-1/\mu } \{ d(\delta/\mu+1) (1+1/a )^{\mu'} \}^{1/\mu} \} } \,d\xi. \end{aligned}$$

We note that if $\mu\neq1$, i.e., $0<\mu<1$, there exists $L>0$ such that

4 $$\begin{aligned} \biggl( \vert a\xi \vert +\frac{1}{ \vert a\xi \vert ^{\mu /\delta}} \biggr)^{1 /\mu} =& \frac{1}{ \vert a\xi \vert ^{1 /\delta}} \bigl( \vert a\xi \vert ^{1+\mu/\delta} +1 \bigr)^{1 /\mu} \\ \leq& \textstyle\begin{cases} \frac{1}{ \vert a\xi \vert ^{1 /\delta}} ( ( \vert a\xi \vert ^{1+\mu/\delta})^{1 /\mu} +1^{1 /\mu}+L \vert a\xi \vert ^{1 /\mu-1} )& \text{if } \vert a\xi \vert \geq1 , \\ \frac{1}{ \vert a\xi \vert ^{1 /\delta}} ( ( \vert a\xi \vert ^{1+\mu/\delta})^{1 /\mu} +1^{1 /\mu}+L \vert a\xi \vert )& \text{if } 0 < \vert a\xi \vert < 1 \end{cases}\displaystyle \\ \leq&\frac{1}{ \vert a\xi \vert ^{1 /\delta}} \bigl( \bigl( \vert a\xi \vert ^{1+\mu/\delta} \bigr)^{1 /\mu} +1^{1 /\mu}+L \vert a\xi \vert ^{\max\{ 1 /\mu-1,1 \} } \bigr) \\ = & \vert a\xi \vert ^{1/\mu } + \frac{1}{ \vert a\xi \vert ^{1/\delta } } +L \vert a\xi \vert ^{k } . \end{aligned}$$

If $\mu=1$, (4) also holds with $L=0$. Lemma 3.1 with $\alpha= \vert \xi \vert $, $\beta= \vert a\xi \vert +1/ \vert a\xi \vert ^{\mu/\delta} $ gives

$$\begin{aligned}& \vert \xi \vert ^{1/\mu} + \vert a\xi \vert ^{1/\mu} + \frac{1}{ \vert a\xi \vert ^{1 /\delta}} - 2^{1-1/\mu} \biggl\{ d(\delta/\mu+1) \biggl(1+ \frac{1}{a} \biggr)^{\mu'} \biggr\} ^{1/\mu} \\& \quad \geq \vert \xi \vert ^{1/\mu} + \biggl( \vert a\xi \vert + \frac{1}{ \vert a\xi \vert ^{\mu/\delta}} \biggr)^{1 /\mu}- L \vert a\xi \vert ^{k } - 2^{1-1/\mu} \biggl\{ d(\delta/\mu+1) \biggl(1+\frac{1}{a} \biggr)^{\mu'} \biggr\} ^{1/\mu} \\& \quad \geq { 2^{1-1/\mu} \biggl( \vert \xi \vert + \vert a \xi \vert + \frac{1}{ \vert a\xi \vert ^{\mu/\delta}} \biggr)^{1 /\mu} -L \vert a\xi \vert ^{k} - 2^{1-1/\mu} \biggl\{ d(\delta/\mu+1) \biggl(1+ \frac{1}{a} \biggr)^{\mu'} \biggr\} ^{1/\mu} } \\& \quad = -L \vert a\xi \vert ^{k } , \end{aligned}$$

here we used

$$\min_{\xi\in\mathbf{R}} \biggl( \vert \xi \vert + \vert a\xi \vert + \frac{1}{ \vert a\xi \vert ^{\mu/\delta} } \biggr) = d(\delta /\mu+1) \biggl(1+\frac{1}{a} \biggr)^{\mu'}. $$

There exist $R \geq1 > r >0$ independent of $a>0$ such that

$$Lh' \vert a\xi \vert ^{k} \leq \bigl(h-h' \bigr) \bigl( \vert a\xi \vert ^{1 /\mu} + \vert a\xi \vert ^{-1 /\delta} \bigr) \quad \text{for } \vert a\xi \vert \geq R \text{ or } \vert a\xi \vert < r, $$

since $-1 /\delta< k= \max\{ 1 /\mu-1, 1\}-1/\delta<1 /\mu$. Therefore, putting

$$I:= \bigl\{ \xi\in\mathbf{R}; r /a \leq \vert \xi \vert < R /a \bigr\} , $$

we have

$$\begin{aligned}& \bigl\vert e^{ h' 2^{1-1/\mu} \{ d(\delta/\mu+1) (1+1/a )^{\mu'}\} ^{1/\mu} }W_{\psi}f (a,b) \bigr\vert \\& \quad \leq Ca^{1/2} \bigl\Vert e^{h \vert \xi \vert ^{1 /\mu }}\hat{f} \bigr\Vert \int_{\mathbf{R}} e^{-(h-h')( \vert \xi \vert ^{1/\mu} + \vert a\xi \vert ^{1 /\mu} + \vert a\xi \vert ^{-1 /\delta}) +Lh' \vert a\xi \vert ^{k} } \,d\xi \\& \quad \leq Ca^{1/2} \bigl\Vert e^{h \vert \xi \vert ^{1 /\mu }}\hat{f} \bigr\Vert \int_{\mathbf{R} \backslash I } e^{-(h-h') \vert \xi \vert ^{1/\mu} } \,d\xi \\& \quad\quad{} + Ca^{1/2} \bigl\Vert e^{h \vert \xi \vert ^{1 /\mu}}\hat{f} \bigr\Vert \int_{I} e^{-(h-h')( \vert \xi \vert ^{1/\mu} + \vert a\xi \vert ^{1 /\mu} + \vert a\xi \vert ^{-1 /\delta}) +Lh' \vert a\xi \vert ^{k} } \,d\xi \\& \quad \leq Ca^{1/2} \bigl\Vert e^{h \vert \xi \vert ^{1 /\mu }}\hat{f} \bigr\Vert \int_{\mathbf{R}} e^{-(h-h') \vert \xi \vert ^{1/\mu} } \,d\xi \\& \quad\quad{} + Ca^{1/2} \bigl\Vert e^{h \vert \xi \vert ^{1 /\mu}}\hat{f} \bigr\Vert \int_{\mathbf{R}} e^{-(h-h') \vert \xi \vert ^{1/\mu} +Lh'\max\{ R^{k} , r^{k} \} } \,d\xi \\& \quad \leq { C a^{1/2} \bigl\Vert e^{h \vert \xi \vert ^{1 /\mu }}\hat{f} \bigr\Vert . } \end{aligned}$$

Thus, it follows that

$$\begin{aligned} \bigl\Vert a^{-1/2} e^{ h' 2^{1-1/\mu} d(\delta/\mu+1)^{1/\mu} a ^{-1/(\mu+\delta)} } W_{\psi}f \bigr\Vert \leq& \bigl\Vert a^{-1/2} e^{ h' 2^{1-1/\mu} \{ d(\delta/\mu+1) (1+1/a )^{\mu'} \}^{1/\mu} } W_{\psi}f \bigr\Vert \\ \leq& C \bigl\Vert e^{h \vert \xi \vert ^{1 /\mu}}\hat{f} \bigr\Vert . \end{aligned}$$

• Case of $0<\mu\leq1$ with the condition $\vert \hat{f} (\xi) \vert \leq C e^{-h \vert \xi \vert ^{-1 /\delta}}$) For $h>h'>0$, we get

$$\begin{aligned}& \bigl\vert e^{ h' 2^{1-1/\mu} \{ d(\delta/\mu+1) (1+a )^{\mu'} (1+1/a^{\mu/\delta})^{\delta'} \}^{1/\mu} }W_{\psi}f (a,b) \bigr\vert \\& \quad \leq Ca^{1/2} \bigl\Vert e^{h ( \vert \xi \vert ^{1 /\mu}+ \vert \xi \vert ^{-1 /\delta})}\hat{f} \bigr\Vert \\& \quad \quad{} \times \int^{\infty}_{-\infty} e^{ -(h-h')\{ \vert \xi \vert ^{1/\mu} + \vert \xi \vert ^{-1 /\delta}+ \vert a\xi \vert ^{1 /\mu} + \vert a\xi \vert ^{-1 /\delta}\}} \\& \quad \quad{} \times e^{-h' \{ \vert \xi \vert ^{1/\mu} + \vert \xi \vert ^{-1 /\delta} + \vert a\xi \vert ^{1 /\mu } + \vert a\xi \vert ^{-1 /\delta} -2^{1-1/\mu} \{ d(\delta/\mu +1) (1+a )^{\mu'} (1+1/a^{\mu/\delta})^{\delta'} \}^{1/\mu} \} } \,d\xi. \end{aligned}$$

By (4) Lemma 3.1 with $\alpha= \vert \xi \vert +1/ \vert \xi \vert ^{\mu/\delta} $, $\beta= \vert a\xi \vert +1/ \vert a\xi \vert ^{\mu/\delta} $ gives

$$\begin{aligned}& \vert \xi \vert ^{1/\mu} + \vert \xi \vert ^{-1 /\delta}+ \vert a\xi \vert ^{1/\mu} +\frac{1}{ \vert a\xi \vert ^{1 /\delta}} - 2^{1-1/\mu} \biggl\{ d(\delta/\mu+1) (1+ a )^{\mu'} \biggl(1+\frac{1}{a^{\mu /\delta} } \biggr)^{\delta'} \biggr\} ^{1/\mu} \\& \quad \geq \biggl( \vert \xi \vert +\frac{1}{ \vert \xi \vert ^{\mu/\delta}} \biggr)^{1 /\mu}+ \biggl( \vert a\xi \vert +\frac{1}{ \vert a\xi \vert ^{\mu /\delta}} \biggr)^{1 /\mu} -L \bigl( \vert \xi \vert ^{k} + \vert a\xi \vert ^{k} \bigr) \\& \quad\quad{} - 2^{1-1/\mu} \biggl\{ d(\delta/\mu+1) (1+ a )^{\mu'} \biggl(1+\frac{1}{a^{\mu/\delta} } \biggr)^{\delta'} \biggr\} ^{1/\mu} \\& \quad \geq 2^{1-1/\mu} \biggl( \vert \xi \vert +\frac{1}{ \vert \xi \vert ^{\mu/\delta}}+ \vert a \xi \vert +\frac{1}{ \vert a\xi \vert ^{\mu/\delta}} \biggr)^{1 /\mu} -L \bigl( \vert \xi \vert ^{k} + \vert a\xi \vert ^{k} \bigr) \\& \quad\quad{} - 2^{1-1/\mu} \biggl\{ d(\delta/\mu+1) (1+ a )^{\mu'} \biggl(1+\frac{1}{a^{\mu/\delta} } \biggr)^{\delta'} \biggr\} ^{1/\mu} \\& \quad = -L \bigl( \vert \xi \vert ^{k} + \vert a\xi \vert ^{k} \bigr) , \end{aligned}$$

here we used

$$\min_{\xi\in\mathbf{R}} \biggl( \vert \xi \vert +\frac{1}{ \vert \xi \vert ^{\mu/\delta} }+ \vert a\xi \vert +\frac{1}{ \vert a\xi \vert ^{\mu/\delta} } \biggr) =d(\delta/\mu+1) (1+ a )^{\mu'} \biggl(1+\frac{1}{a^{\mu/\delta} } \biggr)^{\delta'}. $$

There exist $R, \tilde{R} \geq1> r, \tilde{r} >0 $ independent of $a>0$ such that

$$Lh' \vert a\xi \vert ^{k} \leq \bigl(h-h' \bigr) \bigl( \vert a\xi \vert ^{1 /\mu} + \vert a\xi \vert ^{-1 /\delta} \bigr) \quad \text{for } \vert a\xi \vert >R \text{ or } \vert a\xi \vert < r, $$

and for $\varepsilon>0$ satisfying $h>h'+\varepsilon>h'>0$ (e.g., $\varepsilon= (h-h')/2$)

$$Lh' \vert \xi \vert ^{k} \leq \bigl(h-h'- \varepsilon \bigr) \bigl( \vert \xi \vert ^{1 /\mu} + \vert \xi \vert ^{-1 /\delta} \bigr) \quad \text{for } \vert \xi \vert >\tilde{R} \text{ or } \vert \xi \vert < \tilde{r} , $$

since $-1 /\delta< k=\max\{ 1 /\mu-1, 1\}-1/\delta<1 /\mu$. Therefore, putting

$$I:= \bigl\{ \xi\in\mathbf{R}; r /a \leq \vert \xi \vert < R /a \bigr\} \quad \text{and} \quad J:= \bigl\{ \xi\in\mathbf{R}; \tilde{r} \leq \vert \xi \vert < \tilde{R} \bigr\} , $$

we have

$$\begin{aligned}& \bigl\vert e^{ h' 2^{1-1/\mu}\{ d(\delta/\mu+1) (1+a )^{\mu'} (1+1/a^{\mu/\delta})^{\delta'} \}^{1/\mu} }W_{\psi}f (a,b) \bigr\vert \\ & \quad \leq Ca^{1/2} \bigl\Vert e^{h ( \vert \xi \vert ^{1 /\mu}+ \vert \xi \vert ^{-1 /\delta})}\hat{f} \bigr\Vert \\ & \quad \quad{}\times \int_{\mathbf{R}} e^{-(h-h')( \vert \xi \vert ^{1/\mu} + \vert \xi \vert ^{-1 /\delta}+ \vert a\xi \vert ^{1 /\mu} + \vert a\xi \vert ^{-1 /\delta}) +Lh'( \vert \xi \vert ^{k} + \vert a\xi \vert ^{k} ) } \,d\xi \\ & \quad \leq Ca^{1/2} \bigl\Vert e^{h ( \vert \xi \vert ^{1 /\mu}+ \vert \xi \vert ^{-1 /\delta})}\hat{f} \bigr\Vert \biggl\{ \int_{\mathbf{R} \backslash(I\cup J) } e^{- \varepsilon ( \vert \xi \vert ^{1/\mu} + \vert \xi \vert ^{-1/\delta} ) } \,d\xi \\ & \quad\quad{} + \int_{I\backslash J } e^{- \varepsilon ( \vert \xi \vert ^{1/\mu } + \vert \xi \vert ^{-1/\delta} ) -(h-h')( \vert a\xi \vert ^{1 /\mu} + \vert a\xi \vert ^{-1 /\delta}) +Lh' \vert a\xi \vert ^{k} } \,d\xi \\ & \quad\quad{} + \int_{ J\backslash I } e^{-(h-h')( \vert \xi \vert ^{1/\mu} + \vert \xi \vert ^{-1 /\delta} ) +Lh' \vert \xi \vert ^{k} } \,d\xi \\ & \quad\quad{} + \int_{I \cap J } e^{-(h-h')( \vert \xi \vert ^{1/\mu} + \vert \xi \vert ^{-1 /\delta}+ \vert a\xi \vert ^{1 /\mu} + \vert a\xi \vert ^{-1 /\delta}) +Lh'( \vert \xi \vert ^{k} + \vert a\xi \vert ^{k} ) } \,d\xi \biggr\} \\ & \quad \leq Ca^{1/2} \bigl\Vert e^{h ( \vert \xi \vert ^{1 /\mu}+ \vert \xi \vert ^{-1 /\delta})}\hat{f} \bigr\Vert \biggl\{ \int_{\mathbf{R}} e^{- \varepsilon( \vert \xi \vert ^{1/\mu} + \vert \xi \vert ^{-1/\delta} ) } \,d\xi \\ & \quad\quad{} + \int_{\mathbf{R}} e^{-\varepsilon( \vert \xi \vert ^{1/\mu} + \vert \xi \vert ^{-1/\delta} ) +Lh'\max\{ R^{k} , r^{k} \} } \,d\xi \\ & \quad\quad{} + \int_{\mathbf{R}} e^{-(h-h') ( \vert \xi \vert ^{1/\mu} + \vert \xi \vert ^{-1/\delta} ) +Lh'\max\{ \tilde{R}^{k} ,\tilde{r}^{k} \} } \,d\xi \\ & \quad\quad{} + \int_{\mathbf{R}} e^{-(h-h') ( \vert \xi \vert ^{1/\mu} + \vert \xi \vert ^{-1/\delta} ) +Lh'\max\{ R^{k} ,\tilde{R}^{k} , r^{k} , \tilde{r}^{k} \} } \,d\xi \biggr\} \\ & \quad \leq { C a^{1/2} \bigl\Vert e^{h ( \vert \xi \vert ^{1 /\mu}+ \vert \xi \vert ^{-1 /\delta})}\hat{f} \bigr\Vert . } \end{aligned}$$

Thus, by (3) it follows that for $h >h'>0$,

$$\begin{aligned}& \bigl\Vert a^{-1/2} e^{ h 2^{1-1/\mu}d(\delta/\mu+1)^{1/\mu}( \max\{ a , a^{-1}\})^{1/(\mu+\delta)} } W_{\psi}f \bigr\Vert \\ & \quad \leq \bigl\Vert a^{-1/2} e^{ h' 2^{1-1/\mu}\{ d(\delta/\mu+1) (1+a )^{\mu'} (1+1/a^{\mu/\delta})^{\delta'}\}^{1/\mu} } W_{\psi}f \bigr\Vert \leq C \bigl\Vert e^{h ( \vert \xi \vert ^{1 /\mu }+ \vert \xi \vert ^{-1 /\delta})}\hat{f} \bigr\Vert _{.} \end{aligned}$$

This concludes the proof of Theorem 2.4.

## Concrete examples

In this section, we introduce concrete examples according to whether the order of vanishing moments is finite or infinite.

• *Case of finite vanishing moments*) Let us consider the function and the wavelet

$$f(x) = \operatorname {sech}(h x), \quad \quad\psi(x) =\frac{d }{dx} \operatorname {sech}(h x). $$

In particular, when $h = \sqrt{\frac{\pi}{2}}$, it holds that $\hat{f}( \xi) = f(\xi)$, and we also see that $\hat{\psi}( \xi)=i\xi f(\xi) $ and $\psi\in S^{1, \infty}_{1,h' }(\mathbf{R}) $ with $0< h' <h$. By the change of variables $t =e^{2hax}$, we have the wavelet transform

$$\begin{aligned} W_{\psi}f (a,b) = &\sqrt{\frac{a }{ C_{\psi}}} \int_{\mathbf{R}} \psi(x)f(ax+b) \,dx \\ = &-4h\sqrt{\frac{a }{ C_{\psi}}} \int_{\mathbf{R}} \frac{e^{hx} -e^{-hx}}{(e^{hx} +e^{-hx})^{2}}\cdot\frac {1}{e^{h(ax+b) } +e^{-h(ax+b)} } \,dx \\ = &- \frac{2 }{e^{hb} \sqrt{C_{\psi}a}} \int_{0}^{\infty}\frac{t^{1/a}-1}{(t^{1/a}+1)^{2}} \cdot \frac{dt}{t^{\frac{a-1}{2a} } (t +e^{-2hb})} , \end{aligned}$$

where

$$C_{\psi}= \int_{\mathbf{R}} \frac{ \vert \hat{\psi}(\xi) \vert ^{2}}{ \vert \xi \vert } \,d\xi\quad ( =2\log2 ) . $$

Using the Hölder inequality $A +B \geq\frac {p}{(p-1)^{1-1/p}}A^{1/p}B^{1-1/p}$ with

$$1< \frac{2}{1+ \frac{h' }{h } }< p_{\pm}:=\frac{2}{1\pm\frac{h' }{h\max \{1,a\} } } < \frac{2}{1- \frac{h' }{h } } < \infty \quad \text{for } \operatorname {sgn}b =\pm1 \text{ respectively} , $$

we obtain the estimate (from above)

5 $$\begin{aligned}& \bigl\vert W_{\psi}f (a,b) \bigr\vert \\& \quad \leq \frac{2 }{e^{hb} \sqrt {C_{\psi}a}} \int_{0}^{\infty}\frac{1}{t^{1/a}+1 } \cdot \frac{dt}{t^{\frac{a-1}{2a} } \cdot\frac{p_{\pm}}{(p_{\pm}-1)^{1-1/ p_{\pm}}} t^{1/p_{\pm}} e^{-2hb(1-1/p_{\pm})}} \\& \quad = \frac{2 (p_{\pm}-1)^{1-1/p_{\pm}}e^{ hb(1-2/p_{\pm})} }{p_{\pm}\sqrt {C_{\psi}a}} \int_{0}^{\infty}\frac{1}{t^{1/a}+1 } \cdot \frac{dt}{t^{\frac{a-1}{2a} +\frac{1}{p_{\pm}} } } \\& \quad \leq \frac{C_{h' }e^{ hb(1-2/ p_{\pm})} }{ \sqrt{ a}} \int_{0}^{\infty}\frac{1}{t^{1/a}+1 } \cdot \frac{dt}{t^{ 1- \frac{1}{2a} \pm\frac{h' }{2h \max\{1,a\} } }} \\& \quad \leq \frac{C_{h' }e^{ hb(1-2/ p_{\pm})} }{ \sqrt{ a}} \biggl\{ \int_{0}^{1}\frac{1}{0+1 } \cdot \frac{dt}{t^{ 1- \frac{1}{2a} \pm\frac{h }{2h\max\{1,a\} } }} + \int_{1}^{\infty}\frac{1}{t^{1/a}+0 } \cdot \frac{dt}{t^{ 1- \frac{1}{2a} \pm\frac{h' }{2h \max\{1,a\} } }} \biggr\} \\& \quad = \frac{C_{h' }e^{ hb(1-2/ p_{\pm})} }{ \sqrt{ a}} \biggl\{ \frac {1}{\frac{1}{2a} \pm\frac{h' }{2h \max\{1,a\} } } +\frac{1}{\frac {1}{2a} \mp\frac{h' }{2h \max\{1,a\} }} \biggr\} \\& \quad = \frac{C_{h' }e^{ hb(1-2/ p_{\pm})} }{ \sqrt{ a}} \frac{4a }{1- ( \frac{h' a }{h \max\{1,a\} })^{2}} \\& \quad \leq C_{ h' }a^{1/2}e^{ -h' \vert b \vert / \max\{1,a\} } , \end{aligned}$$

here we used the fact that

$$1- \frac{1}{2a} \pm\frac{h' }{2h \max\{1,a\} } < 1 , \quad \quad 1+\frac {1}{2a} \pm \frac{h' }{2h \max\{1,a\} } >1. $$

Then (i)′ and (ii)′ in Theorem 2.4 become

$$\begin{aligned}& (\mathrm{i})' \quad \bigl\Vert e^{ h' \vert b/(a+1) \vert } W_{\psi}f \bigr\Vert _{L^{\infty}(\mathbf{R}_{+} \times \mathbf{R}) } \leq C \bigl\Vert e^{h \vert x \vert }f \bigr\Vert _{L^{\infty}(\mathbf{R}) }, \\& (\mathrm{ii})'\quad \bigl\Vert a^{-1/2} e^{ h' } W_{\psi}f \bigr\Vert _{L^{\infty}(\mathbf{R}_{+} \times \mathbf{R}) } \leq C \bigl\Vert e^{h \vert \xi \vert }\hat{f} \bigr\Vert _{L^{\infty}(\mathbf{R}) } . \end{aligned}$$

From estimate (5) it is possible that this example is the near critical case of $(\mathrm{i})'$ and $(\mathrm{ii})'$ since $\vert b/(a+1) \vert \sim \vert b \vert / \max\{1,a\} $.

### Remark 4.1

If we consider the typical example of the Mexican hat wavelet

$$\psi(x) =\frac{2}{\pi^{1/4}\sqrt{3}} \bigl(1 -x^{2} \bigr) e^{-x^{2}/2}, \quad \quad\hat{\psi}(\xi) =\frac{2\sqrt{2\pi}}{\pi^{1/4}\sqrt{3}}\xi^{2} e^{-\xi^{2}/2}, $$

we see that $\psi\in S^{1/2, \infty}_{1/2,h'}(\mathbf{R}) $ with $0< h' <h=1/2$. In particular, when $f(x) =e^{-x^{2}/2}\in S^{1/2, \infty}_{1/2,h}(\mathbf {R}) $, the wavelet transform is computed as

$$W_{\psi}f (a,b) =\frac{2\sqrt{2}\pi^{1/4}a^{5/2}(a^{2}-1-b^{2})}{\sqrt{3 C_{\psi}}(a^{2}+1)^{5/2}}e^{-b^{2}/(2a^{2}+2)}. $$

Then (i)′ in Theorem 2.4 becomes

$$(\mathrm{i})' \quad \bigl\Vert e^{ h' 2^{-1} \vert b/(a+1) \vert ^{2} } W_{\psi}f \bigr\Vert _{L^{\infty}(\mathbf{R}_{+} \times \mathbf{R}) } \leq C \bigl\Vert e^{h \vert x \vert ^{2}}f \bigr\Vert _{L^{\infty}(\mathbf{R}) }. $$

The exponent $-b^{2}/(2a^{2}+2)$ is not a critical case of $(\mathrm{i})'$ with $0< h' <h=\frac{1}{2}$ since $h'2^{-1} \vert b/(a+1) \vert ^{2} \sim b^{2}/(4a^{2}+4)$. Therefore, we gave the new wavelet $\psi(x) =\frac{d }{dx} \operatorname {sech}(h x) \in S^{1, \infty}_{1,h' }(\mathbf{R}) $ with $0< h' <h= \sqrt{\frac{\pi}{2}}$.

• *Case of infinite vanishing moments*) Firstly we prove the following.

### Proposition 4.2

*The inverse Fourier transform of* $e^{-\xi^{2}-t^{2}\xi^{-2}}$ *is given by*

6 $$ {\mathcal {F}}^{-1} \bigl[e^{-\xi^{2}-t^{2}\xi^{-2}} \bigr](x) = \frac{1}{ \sqrt{2}} \sum_{n=0}^{\infty}\frac{(-2t)^{n}}{n!} {}_{1}F_{1} \biggl(\frac{1-n}{2}, \frac{1}{2}, -\frac{x^{2}}{4} \biggr), $$

*where* ${}_{1}F_{1} (a,b,z )$ *is the confluent hypergeometric function of the first kind*.

### Remark 4.3

The change of variables also yields

$$\begin{aligned} {\mathcal {F}}^{-1} \bigl[e^{-\xi^{2}/2-t^{2}\xi^{-2}} \bigr](x) =& \sqrt{ \frac{2}{\pi}} \int^{\infty}_{0}e^{-\xi^{2}/2-t^{2}\xi^{-2}}\cos x\xi \,d\xi \\ =& \frac{2}{\sqrt{\pi}} \int^{\infty}_{0}e^{-\xi^{2}-(t/\sqrt{2})^{2}\xi^{-2}}\cos(\sqrt{2}x)\xi \,d \xi \\ = & \sqrt{2} {\mathcal {F}}^{-1} \bigl[e^{-\xi^{2}-(t/\sqrt{2})^{2}\xi^{-2}} \bigr](\sqrt {2}x) \\ = & \sum_{n=0}^{\infty}\frac{(-\sqrt{2} t)^{n}}{n!} {}_{1}F_{1} \biggl(\frac{1-n}{2},\frac{1}{2}, - \frac{x^{2}}{2} \biggr). \end{aligned}$$

### Proof of Proposition 4.2

Let us put

$$I(t,x):={\mathcal {F}}^{-1} \bigl[e^{-\xi^{2}-t^{2}\xi^{-2}} \bigr](x) = \sqrt{ \frac{2}{\pi}} \int^{\infty}_{0}e^{-\xi^{2}-t^{2}\xi^{-2}}\cos x\xi \,d\xi. $$

Differentiating $I(t,x)$ in *x*, we have

$$\begin{gathered} \partial_{x} I(t,x)=- \sqrt{\frac{2}{\pi}} \int^{\infty}_{0} e^{-\xi^{2}-t^{2}\xi^{-2}}\xi\sin x\xi \,d \xi, \\ \partial_{x}^{2} I(t,x)=- \sqrt{\frac{2}{\pi}} \int^{\infty}_{0} e^{-\xi^{2}-t^{2}\xi^{-2}}\xi^{2} \cos x\xi \,d\xi. \end{gathered} $$

On the other hand, differentiating *I* in *t*, we also have

$$\partial_{t} I(t,x)=- \sqrt{\frac{2}{\pi}} 2t \int^{\infty}_{0} e^{-\xi^{2}-t^{2}\xi^{-2}}\xi^{-2} \cos x\xi \,d\xi. $$

Moreover, the integration by parts yields

$$\begin{aligned} \partial_{t} I(t,x) =&- \sqrt{\frac{2}{\pi}} t^{-1} \int^{\infty}_{0} \bigl\{ e^{ -t^{2}\xi^{-2}} \bigr\} 'e^{-\xi^{2} }\xi\cos x\xi \,d\xi \\ =& \sqrt{\frac{2}{\pi}} t^{-1} \int^{\infty}_{0} e^{ -\xi^{2}-t^{2}\xi^{-2}} \bigl\{ \bigl(1-2 \xi^{2} \bigr) \cos x\xi- x\xi\sin x\xi \bigr\} \,d\xi. \end{aligned}$$

Thus, we see that $I(t,x)$ satisfies the partial differential equation

7 $$ \partial_{t} I (t,x) = 2 t^{-1} \biggl\{ \frac{1}{2} I (t,x) +\partial_{x}^{2} I (t,x) + \frac{x}{2}\partial_{x} I (t,x) \biggr\} . $$

We may suppose that $x \geq0$ since $I(t,x) = \sqrt{\frac{2}{\pi}} \int^{\infty}_{0}e^{-\xi^{2}-t^{2}\xi^{-2}}\cos x\xi \,d\xi$ is an even function in *x*. Now we consider the point $x=2\sqrt{-y} $ ($y \leq0$) and get for $J(t,y):=I(t, 2\sqrt{-y})$

$$\partial_{y}J(t,y) =\frac{-1}{ \sqrt{-y}}(\partial_{x}I) (t, 2\sqrt{-y}), \quad\quad \partial^{2}_{y}J(t,y) =- \frac{1}{ y} \bigl(\partial_{x}^{2}I \bigr) (t,2 \sqrt{-y})-\frac{1}{2y }\partial_{y}J(t,y) . $$

Therefore, by the change of variables $x=2\sqrt{-y} $, it holds that

$$\partial_{t} J(t,y) = 2 t^{-1} \biggl\{ \frac{1}{2} J(t,y) -y\partial_{y}^{2} J(t,y) + \biggl(y- \frac{1}{2} \biggr) \partial_{y} J(t,y) \biggr\} . $$

To solve this partial differential equation, we shall use the method of separation of variables. By putting $J (t,y)= \sum_{n =0}^{\infty}L_{n}(t)K_{n}(y)$, we obtain

$$\frac{t\partial_{t} L_{n}(t)}{2L_{n}(t)}=\frac{-y \partial^{2}_{y}K_{n}(y) - (\frac {1}{2} -y ) \partial_{y} K_{n}(y)+\frac{1}{2}K_{n}(y)}{K_{n}(y)}=:\lambda_{n}. $$

We immediately see that $L_{n}(t) =t^{2\lambda_{n} }L_{n}(1)$. It is known that

8 $$ \begin{aligned}[b] I(t,0)&= \sqrt{\frac{2}{\pi}} \int^{\infty}_{0}e^{-\xi^{2}-t^{2}\xi^{-2}} \,d\xi \\ &= \frac{1}{ \sqrt{2} } e^{-2 t}\quad \biggl( \equiv\frac{1}{ \sqrt{2} } \biggl\{ 1 +\frac{(-2)}{1!}t^{1}+\frac{(-2)^{2}}{2!}t^{2}+\cdots \biggr\} \biggr). \end{aligned} $$

We note that

$$I(t,0)=J (t,0) = \sum_{n =0}^{\infty}L_{n}(t)K_{n}(0)= \sum_{n =0}^{\infty}L_{n}(t) , $$

here we may take $K_{n}(0)=1$ for all $n \in\mathbf{N}$ by choosing the suitable $L_{n}(t)$. Hence we see that $\lambda_{n} =\frac{n}{2}$ and

$$L_{n}(t) =t^{n }L_{n}(1)= \frac{1}{ \sqrt{2} } \frac{(-2)^{n}}{n!} t^{n}. $$

Meanwhile, the eigenvalue problem

$$-y \partial^{2}_{y}K_{n}(y) - \biggl( \frac{1}{2} -y \biggr) \partial_{y} K_{n}(y)+ \frac{1}{2}K_{n}(y)=\frac{n}{2} K_{n} (y) $$

with $K_{n}(0)=1$ has

$$K_{n}(y) = {}_{1}F_{1} \biggl(\frac{1-n}{2}, \frac{1}{2}, y \biggr). $$

Thus it follows that

$$J(t,y)= \sum_{n=0}^{\infty}L_{n}(t)K_{n}(y)= \sum_{n=0}^{\infty}\frac{1}{ \sqrt{2} } \frac{(-2)^{n}}{n!} t^{n} {}_{1}F_{1} \biggl(\frac{1-n}{2},\frac{1}{2}, y \biggr) , $$

which gives

9 $$ I(t,x)= \frac{1}{ \sqrt{2} } \sum_{n=0}^{\infty}t^{n} \frac{(-2)^{n}}{n!} {}_{1}F_{1} \biggl( \frac{1-n}{2},\frac{1}{2}, -\frac{x^{2}}{4} \biggr). $$

We knew that $I(t,x)$ is an even function in advance and supposed that $x\geq0$. The last representation also implies that $I(t,x)$ is an even function in *x*. So, (9) holds for all $x \in\mathbf{R}$.

We have derived (9) by solving the partial differential equation. To avoid confusion, let us denote the solution represented as in (9) by $\tilde{I}(t,x) $. It remains to show the uniqueness of $\tilde{I}(t,x) = \frac{1}{ \sqrt{2} } \sum_{n=0}^{\infty}t^{n} \frac{(-2)^{n}}{n!} {}_{1}F_{1} (\frac{1-n}{2},\frac{1}{2}, -\frac{x^{2}}{4} )$ and $I(t,x)= \sqrt{\frac{2}{\pi}} \int^{\infty}_{0}e^{-\xi^{2}-t^{2}\xi^{-2}}\cos x\xi \,d\xi$ except the case of $t=0$. Instead of $I(t,x)$, we consider for $(s,x) \in(0,\infty) \times \mathbf{R}$

$${\mathcal {I}} (s,x) \bigl(=I(\sqrt {s},x) \bigr)= \sqrt{\frac{2}{\pi}} \int^{\infty}_{0}e^{-\xi^{2}-s\xi^{-2}}\cos x\xi \,d\xi $$

for the differentiation with respect to *s*. Then, by Stirling’s formula, we obtain

$$\begin{aligned} \bigl\vert \partial_{s}^{m}\partial_{x}^{j} {\mathcal {I}} (s,x) \bigr\vert \leq& \sqrt{\frac{2}{\pi}} \int^{\infty}_{0}e^{-\xi^{2}/2}\cdot e^{-s\xi^{-2}}\xi^{-2m}\cdot e^{-\xi^{2}/2} \bigl( \xi^{2} \bigr)^{j/2}\,d\xi \\ \leq& C \sup_{\eta\geq0} e^{-s\eta}\eta^{m} \cdot\sup_{\mu\geq0}e^{-\mu/2}\mu^{j/2} \\ \leq& C e^{-m } \biggl( \frac{m}{s} \biggr)^{m} \cdot e^{-j/2 }j^{j/2} \\ \leq& C r_{s}^{m+j}m!(j!)^{1/2} \quad \bigl(\leq C r_{s}^{m+j}m!j! \bigr). \end{aligned}$$

This implies that ${\mathcal {I}} (s,x)$ is analytic for $(s,x) \in [s_{0},\infty) \times\mathbf{R}$ with arbitrarily fixed ${s_{0}>0}$. Therefore, we see that $I (t,x)= \sqrt{\frac{2}{\pi}} \int^{\infty}_{0}e^{-\xi^{2}-t^{2}\xi^{-2}}\cos x\xi \,d\xi$ is analytic for $(t,x) \in {(0,\infty) \times\mathbf{R}}$. □

### Remark 4.4

Probably $I(t,x)$ would be analytic also at $t=0$. But ${\mathcal {I}} (s,x)$ ($=I(\sqrt {s},x)$) loses the analyticity at $s=0$. Indeed, we find that $I(\sqrt {s},0) = \frac{1}{ \sqrt{2} } e^{-2 \sqrt {s}}= \frac{1}{ \sqrt{2} } \{ 1 +\frac {(-2)}{1!}\sqrt {s} +\frac{(-2)^{2}}{2!}s+\cdots \}$.

The Taylor expansion around a point $t=T>0$ gives

$$I(t,x) \biggl(= \sqrt{\frac{2}{\pi}} \int^{\infty}_{0}e^{-\xi^{2}-t^{2}\xi^{-2}}\cos x\xi \,d\xi \biggr)= \sum_{n\geq0, k\geq0}a_{n,k}(t-T)^{n}x^{2k}, $$

since $I(t,x) $ is an even function in *x*. By (9) we also get another Taylor expansion

$$\tilde{I}(t,x) = \frac{1}{ \sqrt{2} } \sum_{n=0}^{\infty}\bigl\{ (t-T)+T \bigr\} ^{n} \frac{(-2)^{n}}{n!} {}_{1}F_{1} \biggl(\frac{1-n}{2},\frac{1}{2}, -\frac{x^{2}}{4} \biggr) = \sum _{n\geq0, k\geq0}\tilde{a}_{n,k}(t-T)^{n}x^{2k}. $$

Then $U(t,x):= I(t,x)-\tilde{I}(t,x)= \sum_{n\geq0, k\geq0} u_{n,k}(t-T)^{n}x^{2k}$ satisfies

$$\partial_{t} U(t,x) = 2 t^{-1} \biggl\{ \frac{1}{2} U (t,x) +\partial_{x}^{2} U (t,x) +\frac{x}{2} \partial_{x} U(t,x) \biggr\} , $$

and by (8)

$$U(t,0)\equiv0. $$

Therefore, we get $u_{n,0}=0$ for all $n \geq0$ and

10 $$ \sum_{n\geq1, k\geq0}nu_{n,k}t(t-T)^{n-1} x^{2k} = \sum_{n\geq0, k\geq0}(2k+1) \bigl\{ u_{n,k}+4(k+1) u_{n,k+1} \bigr\} (t-T)^{n}x^{2k}, $$

here we used that

$$\partial_{x}^{2} I= \sum_{n\geq0, k\geq1}2k(2k-1)u_{n,k}(t-T)^{n}x^{2k-2}= \sum_{n\geq0, k\geq0}2(k+1) (2k+1)u_{n,k+1}(t-T)^{n}x^{2k} . $$

Moreover, the left-hand side of (10) is changed into

$$\sum_{n\geq1, k\geq0}nu_{n,k}t(t-T)^{n-1} x^{2k} = \sum_{n\geq0, k\geq0} \bigl\{ nu_{n,k}+ (n+1) u_{n+1,k}T \bigr\} (t-T)^{n} x^{2k} . $$

Thus, it holds that

$$nu_{n,k}+ (n+1) u_{n+1,k}T = (2k+1) \bigl\{ u_{n,k}+4(k+1)u_{n,k+1} \bigr\} . $$

Hence, when $u_{n,0}=0$ for all $n \geq0$, we find that $u_{n,1}=0$ for all $n \geq0$, and recursively $u_{n,k}=0$ for all $n \geq0 $ and $k \geq0$. So, we have

$$U(t,x)= \sum_{n\geq0, k\geq0}u_{n,k}(t-T)^{n}x^{2k} \equiv0. $$

This concludes that $I(t,x)$ ($= \sqrt{\frac{2}{\pi}} \int^{\infty}_{0}e^{-\xi^{2}-t^{2}\xi^{-2}}\cos x\xi \,d\xi $) must coincide with $\tilde{I}(t,x)$ ($= \frac{1}{ \sqrt{2} } \sum_{n=0}^{\infty}t^{n} \frac {(-2)^{n}}{n!} {}_{1}F_{1} (\frac{1-n}{2},\frac{1}{2}, -\frac{x^{2}}{4} ) $) for $(t,x) \in(0,\infty) \times\mathbf{R}$. □

As an application of Proposition 4.2, we can compute the Fourier transform and the wavelet transform of concrete functions in the Gelfand-Shilov spaces. So, now let us take $\hat{\psi}(\xi) =\hat{f} (\xi) = e^{-\xi^{2}- \xi^{-2}}$. We see that $\psi, f \in S^{1/2,1/2}_{3/2,h} (\mathbf{R})$ for some $h>0$ since $e^{-\xi^{2}} $ gives $\mu=1/2$ and the Gevrey function $e^{- \xi ^{-2}} $ gives $\delta=1/2$ and $\nu=3/2$ by the Paley-Wiener theorem. Then by (2) it follows that

$$\begin{aligned} W_{\psi}f (a,b) = &2\sqrt{ \frac{a}{ C_{\psi}}} \int^{\infty}_{0}e^{-(1+a^{2})\xi^{2}- (1+ a^{-2}) \xi^{-2}}\cos b\xi \,d\xi \\ =& 2\sqrt{ \frac{a}{ C_{\psi}(1+a^{2})} } \int^{\infty}_{0}e^{-\omega^{2}- (1+a^{-2})(1+a^{2}) \omega^{-2}}\cos \frac{b}{\sqrt{1+a^{2} }}\omega \,d\omega \\ =& \sqrt{ \frac{a}{ C_{\psi}(1+a^{2})} } \int^{\infty}_{-\infty} e^{-\omega^{2}- (a +1/a)^{2} \omega^{-2}}e^{i \frac {b}{\sqrt{1+a^{2} }}\omega} \,d\omega \\ = & \sqrt{ \frac{2\pi a}{ C_{\psi}(1+a^{2})} } {\mathcal {F}}^{-1} \bigl[e^{-\omega^{2}- (a +1/a)^{2} \omega^{-2}} \bigr] \biggl( \frac {b}{\sqrt{1+a^{2} }} \biggr) . \end{aligned}$$

By the Paley-Wiener theorem, we find that for some $\rho>0$

$$\bigl\vert W_{\psi}f (a,b) \bigr\vert \leq Ce^{ -\rho \vert b/ \sqrt{ 1+a^{2}} \vert ^{2/3}} \sim Ce^{ -\rho \vert b/( 1+a ) \vert ^{2/3}} . $$

This implies that the order (i) in Theorem 2.4 is almost optimal with respect to *a* and *b*. Using Proposition 4.2 with $t=1$ and $t = a +1/a $, we have the following.

### Theorem 4.5

*Let* $\hat{\psi}(\xi) =\hat{f} (\xi) = e^{-\xi^{2}- \xi^{-2}}$ *for* $\xi \neq0$ *and* =0 *for* $\xi= 0$. *Then*

$$\psi(x) =f(x) = \frac{1}{ \sqrt{2} } \sum_{n=0}^{\infty}\frac{(-2 )^{n}}{n!} {}_{1}F_{1} \biggl(\frac{1-n}{2}, \frac{1}{2}, -\frac{x^{2}}{4} \biggr) \in S^{1/2,1/2}_{3/2,h}( \mathbf{R}) $$

*for some* $h>0$, *and the wavelet transform is given by*

$$W_{\psi}f (a,b) = \sqrt{ \frac{\pi a}{ C_{\psi}(1+a^{2})} }\sum _{n=0}^{\infty}\frac{\{-2 (a +1/a)\}^{n}}{n!} {}_{1}F_{1} \biggl(\frac{1-n}{2},\frac{1}{2}, -\frac{b^{2}}{4(1+a^{2})} \biggr), $$

*where* ${}_{1}F_{1} (a,b,z )$ *is the confluent hypergeometric function of the first kind*.

### Remark 4.6

Especially when $b=0$, we also find

11 $$ \bigl\vert W_{\psi}f (a,0) \bigr\vert = \sqrt{ \frac{\pi a}{ C_{\psi}(1+a^{2})} }e^{-2 (a +\frac{1}{a})} . $$

Then (iii)′ in Theorem 2.4 becomes

$$\bigl\Vert a^{-1/2} e^{h' 2\max\{ a , a^{-1}\}}W_{\psi}f \Vert _{L^{\infty}(\mathbf{R}_{+} \times \mathbf{R})} \leq C \Vert e^{h\max \{ \vert \xi \vert ^{2}, \vert \xi \vert ^{-2}\} }\hat{f} \bigr\Vert _{L^{\infty}(\mathbf{R}) } . $$

(11) implies that $\max\{ a , a^{-1}\} $ in $(\mathrm{iii})'$ cannot be improved anymore since $h' \sim1$ and

$$h' 2 \max \bigl\{ a , a^{-1} \bigr\} \sim2 \biggl(a + \frac{1}{a} \biggr) . $$

### Remark 4.7

As introduced in Remark 1.1, the Bessel wavelet $\psi(x) $ satisfies $\hat{\psi}(\xi) = e^{-\xi- \xi^{-1}}$ for $\xi> 0$ and $\hat{\psi}(\xi)=0$ for $\xi\leq 0$ belongs to ${\mathcal {S}}^{1,+}_{2}(\mathbf{R})$. Hence, we also see that

$$\psi(x)=\frac{1}{\pi\sqrt{1-ix}}K_{1}(2\sqrt{1-ix})+\frac{1}{\pi\sqrt{1+ix}}K_{1}(2 \sqrt{1+ix}) $$

satisfies $\hat{\psi}(\xi) = e^{- \vert \xi \vert - \vert \xi \vert ^{-1}}$ for $\xi\neq0$ and $\hat{\psi}(\xi)=0$ for $\xi=0$ belongs to ${\mathcal {S}}^{1}_{2}(\mathbf{R})$ and $S^{1 ,1 }_{ 2,h}(\mathbf{R}) $ for some $h>0$.

## Conclusions

In this paper, we consider the Banach spaces of Gelfand-Shilov functions satisfying vanishing moment conditions and study the wavelet transforms. Our contributions are as follows:

1. We derived sharp estimates of the wavelet transforms which are useful for the time-frequency analysis, and stated the continuity properties of the wavelet transforms in Gelfand-Shilov spaces as a corollary.
2. We computed the Fourier transforms and the wavelet transforms of concrete functions in the Gelfand-Shilov spaces. These examples show the optimality of estimates in Theorem 2.4.

## Appendix

Concerned with the inverse wavelet transform, we also get the following.

### Theorem A.1

*Let* *μ*, *ν*, *h*, $h'$ *and* *δ* *be positive constants such that* $\mu+\nu\geq1$, $h'< h$. *Define that* $d(\lambda)= \lambda(\lambda-1)^{-1+1/\lambda} $. *Then*, *for the inverse wavelet transform* $M_{\psi}$ *with the wavelet* $\psi\in S^{\mu,\delta}_{\nu, h}(\mathbf{R}) $, *the following estimates hold*: *for* $F \in V^{ \mu,\delta} _{\nu, h}(\mathbf{R}_{+} \times \mathbf{R}) $

$$\begin{aligned}& (\mathrm{iv}) \quad \bigl\Vert e^{h d(\nu/\mu+1) \vert x \vert ^{1 / (\mu + \nu) }}M_{\psi}F \bigr\Vert _{L^{\infty}( \mathbf{R}) } \\& \hphantom{ (\mathrm{iv}) \quad}\quad \leq C \bigl\Vert e^{h \{ \vert b/\max\{ 1,a\} \vert ^{1 /\nu} +a^{1 /\mu} + a^{-1 /\delta} \} } F \bigr\Vert _{L^{\infty}(\mathbf{R}_{+} \times \mathbf{R}) } \quad \textit{if } \nu>1 , \\& (\mathrm{iv})' \quad \bigl\Vert e^{h'2^{1-1/\nu} d(\nu/\mu+1) \vert x \vert ^{1 / (\mu+ \nu) }}M_{\psi}F \bigr\Vert _{L^{\infty}( \mathbf{R}) } \\& \hphantom{ (\mathrm{iv})' \quad}\quad \leq C \bigl\Vert e^{h \{ \vert b/\max\{ 1,a\} \vert ^{1 /\nu} +a^{1 /\mu} + a^{-1 /\delta} \} } F \bigr\Vert _{L^{\infty}(\mathbf{R}_{+} \times \mathbf{R}) } \quad \textit{if } 0< \nu\leq1 , \\& (\mathrm{v})\quad \biggl\Vert \frac{ \vert \xi \vert ^{1/2} e^{hd(\delta/\mu +1)^{1/\mu} ( \max\{ \vert \xi \vert , \vert \xi \vert ^{- 1}\}) ^{1/(\mu+ \delta) } } }{ \vert \xi \vert +1} { \mathcal {F}}[M_{\psi}F] \biggr\Vert _{L^{\infty}(\mathbf{R} ) } \\& \hphantom{ (\mathrm{v})\quad}\quad \leq \bigl\Vert e^{h \{ \vert b/\max\{ 1,a\} \vert ^{1 /\nu} +a^{1 /\mu} + a^{-1 /\delta} \} } F \bigr\Vert _{L^{\infty}(\mathbf{R}_{+} \times \mathbf{R}) } \quad\textit{if } \mu>1 , \\& (\mathrm{v})' \quad \bigl\Vert e^{h' 2^{1-1/\mu} d(\delta/\mu+1)^{1/\mu} ( \max\{ \vert \xi \vert , \vert \xi \vert ^{- 1}\}) ^{1/(\mu+ \delta) } } { \mathcal {F}}[M_{\psi}F] \bigr\Vert _{ L^{\infty}( \mathbf{ R}) } \\& \hphantom{ (\mathrm{v})' \quad}\quad \leq C \bigl\Vert e^{h \{ \vert b/\max\{ 1,a\} \vert ^{1 /\nu} +a^{1 /\mu} + a^{-1 /\delta} \} } F \bigr\Vert _{L^{\infty}(\mathbf{R}_{+} \times \mathbf{R}) } \quad \textit{if } 0< \mu\leq1 . \end{aligned}$$

The weight function of (iv) and (v) can be estimated as

$$e^{h \{ \vert b/\max\{1,a\} \vert ^{1 /\nu} +a^{1 /\mu} + a^{-1 /\delta} \} } \leq e^{3h \max\{ \vert b/(a+1) \vert ^{1 /\nu}, a^{1 /\mu}, a^{-1 /\delta} \} }, $$

and estimated from below as

$$e^{h d(\nu/\mu+1) \vert x \vert ^{1 / (\mu+ \nu) }}\geq c e^{h \vert x \vert ^{1 / (\mu+ \nu) }}, $$

and

$$\frac{ \vert \xi \vert ^{1/2}e^{hd(\delta/\mu+1)^{1/\mu} ( \max\{ \vert \xi \vert , \vert \xi \vert ^{- 1}\}) ^{1/(\mu+ \delta) } } }{ \vert \xi \vert +1} \geq\frac{ c e^{h \{ \vert \xi \vert ^{1/(\mu+ \delta) } + \vert \xi \vert ^{-1/(\mu+ \delta) } \} }}{ \vert \xi \vert ^{1/2}+ \vert \xi \vert ^{-1/2}} \geq\frac{c}{2} e^{h \{ \vert \xi \vert ^{1/(\mu+ \delta) } + \vert \xi \vert ^{-1/(\mu+ \delta) } \} } $$

in the same way with (1). Therefore, by Theorem A.1, we can also get the following continuity property.

### Corollary A.2

*Let* *μ*, *ν*, *h* *and* *δ* *be constants such that* $\mu>1$, $\nu >1$, $h>0$ *and* $\delta>0$. *Then*, *for the wavelet* $\psi\in S^{\mu,\delta}_{\nu, h}(\mathbf {R}) $, *the inverse wavelet transform* $V^{ \mu, \delta}_{\nu, 3h }(\mathbf{R}_{+} \times \mathbf{R})\ni F \mapsto M_{\psi}F \in S^{\mu+\delta,\mu+\delta}_{\mu+ \nu, h}(\mathbf{R}) $ *is continuous*.

We shall only give a sketch of the proof of Theorem A.1.

• Case of $\nu>1$) From the definition of the inverse wavelet transform we get

$$\begin{aligned}& \bigl\vert e^{h d(\nu/\mu+1) \vert x \vert ^{1 / (\mu+ \nu ) }}M_{\psi}F(x) \bigr\vert \\& \quad \leq C \bigl\Vert e^{h \{ \vert b/\max\{1,a\} \vert ^{1 /\nu} +a^{1 /\mu} + a^{-1 /\delta} \} } F \bigr\Vert \\& \quad \quad{} \times \int_{\mathbf{R}_{+}} \int_{\mathbf{R}}a^{-5/2}e^{ -h \{ \vert b/\max\{1,a\} \vert ^{1 /\nu}+ a^{1 /\mu} + a^{-1 /\delta} + \vert (x-b)/a \vert ^{1/\nu} - d(\nu/\mu+1) \vert x \vert ^{1 / (\mu+ \nu) }\} } \,db\,da. \end{aligned}$$

We shall use the Hölder inequality $A +B \geq (Ap)^{1/p}(Bq)^{1/q}$ with $p= \mu/\nu+1 $, $q = \nu/\mu+1$. If $a\geq1$, Lemma 3.1 with $\alpha= \vert x/a -b/a \vert $, $\beta= \vert b/a \vert $ gives

$$\begin{aligned}& \biggl\vert \frac{b}{\max\{1,a\}} \biggr\vert ^{1 /\nu} + a^{1 /\mu} + a^{-1 /\delta}+ \biggl\vert \frac{x-b}{a} \biggr\vert ^{1/\nu} - d \biggl( \frac{\nu}{\mu}+1 \biggr) \vert x \vert ^{1 / (\mu+ \nu) } \\& \quad \geq \bigl(2 -2^{1/\nu} \bigr)\min \biggl\{ \biggl\vert \frac{x}{a} -\frac{b}{a} \biggr\vert ^{1/\nu} , \biggl\vert \frac{b}{a} \biggr\vert ^{1/\nu} \biggr\} + \biggl\vert \frac{x}{a} \biggr\vert ^{1/\nu} + a^{1 /\mu} - d \biggl( \frac{\nu}{\mu}+1 \biggr) \vert x \vert ^{1 / (\mu+ \nu) } + a^{-1 /\delta} \\& \quad \geq \bigl(2 -2^{1/\nu} \bigr)\min \biggl\{ \biggl\vert \frac{x}{a} -\frac{b}{a} \biggr\vert ^{1/\nu} , \biggl\vert \frac{b}{a} \biggr\vert ^{1/\nu} \biggr\} + a^{-1 /\delta} . \end{aligned}$$

If $0 < a< 1$, Lemma 3.1 with $\alpha= \vert x-b \vert $, $\beta= \vert b \vert $ gives

$$\begin{aligned}& \biggl\vert \frac{b}{\max\{1,a\}} \biggr\vert ^{1 /\nu} + a^{1 /\mu} + a^{-1 /\delta}+ \biggl\vert \frac{x-b}{a} \biggr\vert ^{1/\nu} - d \biggl( \frac{\nu}{\mu}+1 \biggr) \vert x \vert ^{1 / (\mu+ \nu) } \\& \quad \geq \vert b \vert ^{1/\nu} +d \biggl(\frac{\nu}{\mu}+1 \biggr) \vert x-b \vert ^{1 / (\mu+ \nu) } - d \biggl(\frac{\nu}{\mu}+1 \biggr) \vert x \vert ^{1 / (\mu+ \nu) } + a^{-1 /\delta} \\& \quad \geq { d \biggl(\frac{\nu}{\mu}+1 \biggr) \bigl(2 -2^{1/(\mu+ \nu) } \bigr)\min \bigl\{ \vert x-b \vert ^{1/(\mu+ \nu ) } , \vert b \vert ^{1/(\mu+ \nu) } \bigr\} - m_{\mu, \nu} + a^{-1 /\delta} ,} \end{aligned}$$

here we used

$$\vert b \vert ^{1/\nu} -d \biggl(\frac{\nu}{\mu}+1 \biggr) \vert b \vert ^{1 / (\mu+ \nu) }\geq- { ^{\exists}}m_{\mu, \nu} \quad \text{for } b \in\mathbf{R}. $$

Thus, it follows that

$$\begin{aligned} \bigl\Vert e^{h d(\nu/\mu+1) \vert x \vert ^{1 / (\mu+ \nu) }}M_{\psi}F \bigr\Vert \leq& C \bigl\Vert e^{h \{ \vert b/\max\{1,a\} \vert ^{1 /\nu} +a^{1 /\mu} + a^{-1 /\delta} \} } F \bigr\Vert \\ &{} \times C \biggl\{ \int_{1}^{\infty}a^{-3/2}e^{ -h a^{-1 /\delta} } \,da + \int_{0}^{1} a^{-5/2}e^{ -h \{ - m_{\mu, \nu} + a^{-1 /\delta}\} } \,da \biggr\} \\ \leq& C \bigl\Vert e^{h \{ \vert b/\max\{1,a\} \vert ^{1 /\nu } +a^{1 /\mu} + a^{-1 /\delta} \} } F \bigr\Vert . \end{aligned}$$

• Case of $0< \nu\leq1$) This case can be shown similarly as the case of $\nu>1$.

• Case of $\mu>1$) This case can be shown similarly as the case of $\mu> 1$ with the condition $\vert \hat{f}(\xi) \vert \leq C e^{- \vert \xi \vert ^{1/\delta}} $ for the wavelet transform by exchanging the roles of *a* and *ξ*.

• Case of $0< \mu\leq1$) For $h >h'>0$, we get

$$\begin{aligned}& \bigl\vert e^{h' 2^{1-1/\mu} \{ d ( \delta/ \mu+1 ) (1+ \vert \xi \vert )^{\mu'} (1+1/ \vert \xi \vert ^{\mu/\delta} )^{\delta'} \}^{1/\mu}}{ \mathcal {F}}[M_{\psi}F](\xi) \bigr\vert \\& \quad \leq C \bigl\Vert e^{h \{ \vert b/\max\{1,a\} \vert ^{1 /\nu} +a^{1 /\mu} + a^{-1 /\delta} \} } F \bigr\Vert \int_{\mathbf{R}_{+}} \int_{\mathbf{R}}a^{-3/2}e^{ -(h -h') \{ a^{1 /\mu} + a^{-1 /\delta} + \vert a\xi \vert ^{1 /\mu} + \vert a\xi \vert ^{-1 /\delta} \}} \\& \quad\quad{} \times e^{-h ' \{ \vert b/\max\{1,a\} \vert ^{1 /\nu}+ a^{1 /\mu} + a^{-1 /\delta} + \vert a\xi \vert ^{1 /\mu} + \vert a\xi \vert ^{-1 /\delta} - 2^{1-1/\mu} \{ d ( \delta/ \mu+1 ) (1+ \vert \xi \vert )^{\mu'} (1+1/ \vert \xi \vert ^{\mu/\delta} )^{\delta'} \}^{1/\mu} \} } \,db\,da. \end{aligned}$$

Similarly as the case of $0 < \mu\leq1$ for the wavelet transform, by (4) Lemma 3.1 with $\alpha= a + a^{-\mu/\delta}$, $\beta= \vert a\xi \vert + \vert a\xi \vert ^{-\mu/\delta}$ gives

$$\begin{aligned}& \biggl\vert \frac{b}{\max\{1,a\}} \biggr\vert ^{1 /\nu} + a^{1 /\mu} + a^{-1 /\delta}+ \vert a\xi \vert ^{1 /\mu} + \vert a\xi \vert ^{-1 /\delta} \\& \quad\quad{} - 2^{1-1/\mu} \biggl\{ d \biggl( \frac{\delta}{\mu}+1 \biggr) \bigl(1+ \vert \xi \vert \bigr)^{\mu'} \biggl(1+\frac{1}{ \vert \xi \vert ^{\mu/\delta} } \biggr)^{\delta'} \biggr\} ^{1/\mu} \\& \quad \geq { -L \bigl(a^{k}+ \vert a\xi \vert ^{k } \bigr) + \biggl\vert \frac{b}{\max\{1,a\}} \biggr\vert ^{1 /\nu} .} \end{aligned}$$

There exist $R, \tilde{R} \geq1> r, \tilde{r} >0 $ independent of $a>0$ such that

$$Lh' \vert a\xi \vert ^{k} \leq \bigl(h-h' \bigr) \bigl( \vert a\xi \vert ^{1 /\mu} + \vert a\xi \vert ^{-1 /\delta} \bigr) \quad \text{for } \vert a\xi \vert >R \text{ or } \vert a\xi \vert < r, $$

and for $\varepsilon>0$ satisfying $h>h' +\varepsilon>h'>0$ (e.g., $\varepsilon= (h-h')/2$)

$$Lh'a^{k} \leq \bigl(h-h'-\varepsilon \bigr) \bigl( a^{1 /\mu} +a^{-1 /\delta} \bigr) \quad \text{for } a > \tilde{R} \text{ or } a< \tilde{r} , $$

since $-1 /\delta< k=\max\{ 1 /\mu-1, 1\}-1/\delta<1 /\mu$. Therefore, putting

$$I:= \bigl\{ a \in\mathbf{R}_{+}; r / \vert \xi \vert \leq a< R / \vert \xi \vert \bigr\} \quad\text{and} \quad J:= \{ a \in\mathbf{R}_{+}; \tilde{r} \leq a< \tilde{R} \}, $$

we have

$$\begin{aligned}& \bigl\vert e^{h' 2^{1-1/\mu} \{ d ( \delta/ \mu+1 ) (1+ \vert \xi \vert )^{\mu'} (1+1/ \vert \xi \vert ^{\mu/\delta} )^{\delta'} \}^{1/\mu}}{ \mathcal {F}}[M_{\psi}F](\xi) \bigr\vert \\& \quad \leq C \bigl\Vert e^{h \{ \vert b/\max\{1,a\} \vert ^{1 /\nu } +a^{1 /\mu} + a^{-1 /\delta} \} } F \bigr\Vert \\& \quad\quad{} \times \int_{\mathbf{R}_{+}} \min \bigl\{ a^{-1/2}, a^{-3/2} \bigr\} e^{ -(h -h') \{ a^{1 /\mu} + a^{-1 /\delta} + \vert a\xi \vert ^{1 /\mu} + \vert a\xi \vert ^{-1 /\delta} \}+Lh' (a^{k}+ \vert a\xi \vert ^{k }) } \,da \\& \quad \leq C \bigl\Vert e^{h \{ \vert b/\max\{1,a\} \vert ^{1 /\nu} +a^{1 /\mu} + a^{-1 /\delta} \} } F \bigr\Vert \biggl\{ \int_{\mathbf{R}_{+} \backslash(I\cup J) } \min \bigl\{ a^{-1/2}, a^{-3/2} \bigr\} e^{- \varepsilon (a^{1/\mu} +a^{-1/\delta} ) } \,da \\& \quad\quad{} + \int_{I\backslash J } \min \bigl\{ a^{-1/2}, a^{-3/2} \bigr\} e^{- \varepsilon (a^{1/\mu} +a^{-1/\delta} ) -(h-h')( \vert a\xi \vert ^{1 /\mu} + \vert a\xi \vert ^{-1 /\delta}) +Lh' \vert a\xi \vert ^{k} } \,da \\& \quad\quad{} + \int_{ J\backslash I } \min \bigl\{ a^{-1/2}, a^{-3/2} \bigr\} e^{-(h-h')(a^{1/\mu} + a^{-1 /\delta} ) +Lh' a^{k} } \,da \\& \quad\quad{} + \int_{I \cap J } \min \bigl\{ a^{-1/2}, a^{-3/2} \bigr\} e^{-(h-h')( a^{1/\mu} + a^{-1 /\delta}+ \vert a\xi \vert ^{1 /\mu} + \vert a\xi \vert ^{-1 /\delta}) +Lh'( a^{k} + \vert a\xi \vert ^{k} ) } \,da \biggr\} \\& \quad \leq C \bigl\Vert e^{h \{ \vert b/\max\{1,a\} \vert ^{1 /\nu } +a^{1 /\mu} + a^{-1 /\delta} \} } F \bigr\Vert . \end{aligned}$$

Thus, by the inequality $( 1+ \vert \xi \vert )^{\mu'} (1+\frac{1}{ \vert \xi \vert ^{\mu/\delta}} )^{\delta'} \geq ( \max\{ \vert \xi \vert ,\frac{1}{ \vert \xi \vert } \} )^{\mu'}$, it follows that

$$\bigl\Vert e^{h' 2^{1-1/\mu} d(\delta/\mu+1)^{1/\mu} ( \max\{ \vert \xi \vert , \vert \xi \vert ^{- 1}\}) ^{1/(\mu+ \delta) } } { \mathcal {F}}[M_{\psi}F] \Vert \leq \Vert e^{h \{ \vert b/\max\{1,a\} \vert ^{1 /\nu} +a^{1 /\mu} + a^{-1 /\delta} \} } F \bigr\Vert . $$
